# Integrative Single‐Cell Transcriptomics and Epigenomics Mapping of the Fetal Retina Developmental Dynamics

**DOI:** 10.1002/advs.202206623

**Published:** 2023-04-05

**Authors:** Ruonan Li, Jiangyi Liu, Ping Yi, Xianli Yang, Jun Chen, Chenyang Zhao, Xingyun Liao, Xiaotang Wang, Zongren Xu, Huiping Lu, Hongshun Li, Zhi Zhang, Xianyang Liu, Junjie Xiang, Ke Hu, Hongbo Qi, Jia Yu, Peizeng Yang, Shengping Hou

**Affiliations:** ^1^ The First Affiliated Hospital of Chongqing Medical University Chongqing 400016 P. R. China; ^2^ Chongqing Key Laboratory of Ophthalmology Chongqing 400016 P. R. China; ^3^ Chongqing Eye Institute Chongqing 400016 P. R. China; ^4^ Chongqing Branch (Municipality Division) of National Clinical Research Center for Ocular Diseases Chongqing 400016 P. R. China; ^5^ Department of Obstetrics and Gynecology The Third Affiliated Hospital of Chongqing Medical University Chongqing 401120 P. R. China; ^6^ Department of Obstetrics Women and Children's Hospital of Chongqing Medical University Chongqing 401147 P. R. China; ^7^ Department of Medical Oncology Chongqing University Cancer Hospital Chongqing 400030 P. R. China; ^8^ Chongqing Key Laboratory of Maternal and Fetal Medicine The First Affiliated Hospital of Chongqing Medical University Chongqing 400016 P. R. China; ^9^ State Key Laboratory of Medical Molecular Biology Department of Biochemistry and Molecular Biology Haihe Laboratory of Cell Ecosystem Institute of Basic Medical Sciences Chinese Academy of Medical Sciences School of Basic Medicine Peking Union Medical College Beijing 100005 P. R. China; ^10^ The Key Laboratory of RNA and Hematopoietic Regulation Chinese Academy of Medical Sciences Beijing 100005 P. R. China; ^11^ Beijing Institute of Ophthalmology Beijing Tongren Eye Center Beijing Tongren Hospital Capital Medical University Beijing Ophthalmology & Visual Sciences Key Laboratory Beijing 100730 P. R. China

**Keywords:** fetal eye, gliogenesis, neurogenesis, retinal development, single‐cell assay for transposase‐accessible chromatin sequencing, single‐cell RNA sequencing

## Abstract

The underlying mechanisms that determine gene expression and chromatin accessibility in retinogenesis are poorly understood. Herein, single‐cell RNA sequencing and single‐cell assay for transposase‐accessible chromatin sequencing are performed on human embryonic eye samples obtained 9–26 weeks after conception to explore the heterogeneity of retinal progenitor cells (RPCs) and neurogenic RPCs. The differentiation trajectory from RPCs to 7 major types of retinal cells are verified. Subsequently, diverse lineage‐determining transcription factors are identified and their gene regulatory networks are refined at the transcriptomic and epigenomic levels. Treatment of retinospheres, with the inhibitor of RE1 silencing transcription factor, X5050, induces more neurogenesis with the regular arrangement, and a decrease in Müller glial cells. The signatures of major retinal cells and their correlation with pathogenic genes associated with multiple ocular diseases, including uveitis and age‐related macular degeneration are also described. A framework for the integrated exploration of single‐cell developmental dynamics of the human primary retina is provided.

## Graphical abstract

This work applies integrative single‐cell RNA sequencing and single‐cell assay for transposase‐accessible chromatin sequencing to verify the differentiation trajectory from retinal progenitor cells tomajorretinal cell types, identify diverse lineage‐determining transcription factorsand refine gene regulatory networks. The RE1 silencing transcription factor may be involved in the regulation between Müller glial cells and late neurons during embryonic retinal development.

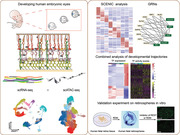

## 1 Introduction

The eye is a vital and highly specialized visual organ, and the retina is the most important component of vision production in the eye. The retina primarily comprises six types of neurons and several types of glial cells. Therein, photoreceptor cells, including cones and rods in the outer nuclear layer (ONL), receive and process light signals from the external environment. The interneurons, including amacrine cells (ACs), bipolar cells (BCs), and horizontal cells (HCs) in the inner plexiform layer (IPL), inner nuclear layer (INL), and outer plexiform layer (OPL), deliver signals from the photoreceptor cells to retinal ganglion cells (RGCs) in the ganglion cell layer (GCL). Inside the RGCs, these light signals are converted to electrical signals and transmitted to the brain.^[^ ^1^ ^]^ The primary types of retinal glial cells include Müller glial cells (MGCs) and microglia. MGCs mainly act as mediators to assist photoreceptor cells in light absorption, provide nutrients to neurons, and remove metabolic waste,^[^ ^2^, ^3^ ^]^ whereas microglia are resident immune cells in the retina and central nervous system that have a critical role in the maintenance of normal homeostasis and immune surveillance of these systems.^[^ ^4^ ^]^

The retina primarily develops from retinal progenitor cells (RPCs), which differentiate into six classes of neurons and one class of glial cells at chronologically separate, yet frequently overlapping, intervals during development. Before becoming terminal cells, RPCs typically transition through a precursor cell stage. Distinct evolutionary fates are selected as the RPCs reach saddle points, where they segregate into different and progressively restricted precursor cell states and finally develop and mature into terminal cells.^[^ ^5^, ^6^, ^7^, ^8^, ^9^ ^]^ Although terminal‐cell specification has received considerable attention, it remains unclear how heterogeneity develops within RPCs.

The general process of retinal development is meticulously regulated by cell‐type‐specific transcription factors (TFs) that recruit chromatin effectors to repurpose the chromatin and promote new retinal cellular characteristics.^[^ ^10^ ^]^ Dysregulation of any step in this process can cause varying degrees of visual dysfunction and congenital diseases, including retinoblastoma, Leber congenital amaurosis, and autosomal recessive retinitis pigmentosa.^[^ ^10^, ^11^, ^12^, ^13^ ^]^ Meanwhile, the development of retinal cell lineages remains to be investigated, and prospective critical factors have not yet been thoroughly characterized.

Recently, the single‐cell assay for transposase‐accessible chromatin sequencing (scATAC‐seq) and single‐cell RNA sequencing (scRNA‐seq) have proven effective for the analysis of human embryonic retinal development. More specifically, scRNA‐seq has been employed to investigate the differentiation trajectory of RPCs and unique subpopulations of embryonic retinal cells. The associated studies have reported that myriad TFs have crucial roles in directing the development of specific lineages, such as nuclear factor I (NFI) in the regulation of cell‐cycle exit and generation of late‐born retinal cell types, and atonal bHLH transcription factor 7 (ATOH7) in the specification of cone photoreceptors.^[^ ^14^, ^15^, ^16^, ^17^ ^]^ Meanwhile, gene expression patterns of important lineage‐defined TFs are primarily regulated by epigenetic programs that are influenced by changes in chromatin accessibility and can be detected by scATAC‐seq. For instance, the sequences of various TF cascades responsible for determining cell fate have been verified at the epigenomic level from a developing human retina database using scATAC‐seq.^[^ ^18^, ^19^, ^20^ ^]^ However, integrated scRNA‐seq and scATAC‐seq datasets from the same human embryonic eye sample are lacking; hence, transcriptomic and epigenetic results have the potential to be better matched.

Here, we performed an integrative analysis of scATAC‐seq and scRNA‐seq on individual cells obtained from human embryonic eyes to capture the dynamic transcriptomic andepigenetic landscapes of the developing human eye at single‐cell resolution. To this end, we probed the precursors of MGCs and the intrinsic connection among neurogenic RPCs (NPCs). We then constructed developmental trajectories and gene regulatory networks (GRNs) for RPC‐derived cells. In this way, we defined the continuous evolution of TF motif activity related to neuronal specification and identified the co‐dependence of TF motif accessibility along these trajectories. By investigating whether RE1 silencing transcription factor (REST) influences RPC and MGC fate, we found that treatment of retinospheres with a REST inhibitor induced neurogenesis with a regular spatial arrangement and decrease in MGCs. Finally, we identified differences between embryonic macrophages and microglia and combined disease‐related genes from retina‐related diseases to characterize relationships between risk factors and specific retinal cell types. Collectively, this study highlights neurogenic cell fate determination processes and elucidates a portion of the gene regulation involved in retinal development.

## 2 Results

### 2.1 Construction of a Single‐Cell Regulatory Atlas of the Developing Human Eye

**Figure 1:**
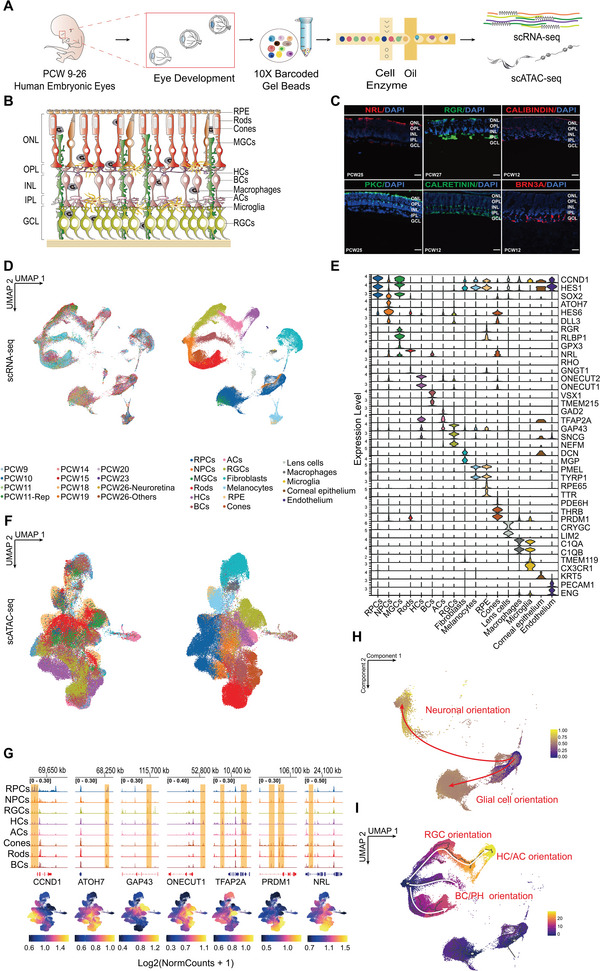
A single‐cell regulatory atlas of the developing human retina. A) Schematic of experimental design. B) Retinal structure schematic. C) Immunofluorescence analysis of primary human retinal tissue validating the expression of cell‐type‐specific markers, including NRL (rods); RGR (MGCs); CALIBINDIN (HCs); PKC (BCs); CALRETININ (HCs, ACs, and RGCs); and BRN3A (RGCs). Nuclei are counterstained with DAPI. Scale bar, 50 µm. D) UMAP embedding of the retina scRNA‐seq dataset, with individual cells colored by age and annotated cell types. E) Violin plot showing relative expression of transcripts with high specificity for individual cell types ordered by cell type. F) UMAP embedding of the retina scATAC‐seq dataset, with individual cells colored by age and annotated cell types. G) Coverage plots and gene score of known marker genes for cell types. Gene color representing the direction of gene translation (red: right; blue: left). H) UMAP embedding of the developmental trajectories of RPCs, MGCs, and NPCs colored by pseudo‐time. Red arrows show two developmental directions from RPCs. I) UMAP embedding of the developmental trajectories of RPCs and RPC‐derived cells from scRNA‐seq colored by pseudo‐time. White arrows show three neuronal lineages. Abbreviations: PCW, post‐conception weeks; RGCs, retinal ganglion cells; HCs, horizontal cells; ACs, amacrine cells; BCs, bipolar cells; MGCs, Müller glial cells; GCL, ganglion cell layer; ONL, outer nuclear layer; OPL, outer plexiform layer; INL, inner nuclear layer; IPL, inner plexiform layer.

To comprehensively characterize chromatin accessibility and gene expression in embryonic eyes, we generated an integrative scATAC‐seq and scRNA‐seq catalog of developing human embryonic eyes at nine time points from postconceptional weeks (PCW) 9 to 26 (PCW9, PCW10, PCW11, PCW14, PCW15, PCW18, PCW20, PCW23, PCW26), including a series of decisive critical retinogenesis events (**Figure** **1**A). Separate scATAC‐seq runs were performed at PCW11 (one biological replicate) and PCW19. Moreover, the PCW26 eyes were divided into “Neuroretina” and “Others” prior to scATAC‐seq and scRNA‐seq analyses. Following initial data processing and quality control, 93527 single‐cell transcriptomes were collected with a median of 2209 genes per cell (Tables S1, Supporting Information). According to recognized marker genes for each retinal cell type, CALRETININ^+^ cells were primarily identified in the OPL, IPL, and GCL, whereas RGR^+^ scaffolding covered the retina during the late‐born stages. Additionally, NRL^+^, PKC^+^, and BRN3A^+^ cells were detected in the ONL, INL, and GCL, respectively, whereas CALBINDIN^+^ cells appeared predominately in the OPL (Figure 1B,C).

Using scRNA‐seq, most cells were divided into 17 groups according to the normalized expression of known markers (Figure 1D,E; Table S2, Supporting Information). Due to the limited number of MGCs (38 cells, 0.0505% of total cell count) and microglia (11 cells, 0.0146% of total cell count) directly mapped to the chromatin landscape in scRNA‐seq, subsequent analysis was not performed (Tables S3 and S4, Supporting Information). We then integrated the gene expression levels with corresponding gene activity scores and found that major cluster annotations in the retina of matched cells were consistent. A group of RPCs expressing CCND1, HES1, and SOX2 was identified, and several NPC markers, including ATOH7, HES6, and DLL3, were expressed by cells in one scRNA‐seq cluster. Additionally, a group of RGCs expressing NEFM, SNCG, and GAP43, as well as genes related to HC identification (ONECUT1 and ONECUT2), was detected. Moreover, a group of cells expressing AC‐related genes (GAD2 and TFAP2A) was observed. Among the BC clusters, a group of cells expressing VSX1 and TMEM215, and distinct clusters of photoreceptor cells, including cones (expressing PRDM1 and THRB) and rods (expressing NRL, RHO) were detected. We further annotated a cluster of cells highly expressing RGR, GPX3, and RLBP1 as MGCs and determined that RGR might be more specific to MGCs than RLBP1 during the embryonic period. Furthermore, we observed gene expression in clusters of retinal pigment epithelia (RPE) (RPE65 and TTR), fibroblasts (DCN and MGP), melanocytes (PMEL and TYRP1), endothelial cells (PECAM1 and ENG), lens cells (CRYGC and LIM2), and corneal epithelium (KRT5). Numerous markers exhibited dynamic gene activity scores in related scATAC‐seq clusters (Figure 1F,G). The emergence of these major retinal cell types reflected normal retinal development (Figure S1A, Supporting Information).

Considering that RPCs have a two‐branch developmental tendency, including glial cell orientation and neuronal orientation,^[^ ^6^, ^14^, ^16^ ^]^ we extracted RPCs, NPCs, and MGCs, as well as six major neurons for pseudo‐time analysis via two different approaches (Palantir and Monocle3). The results verified that RPCs could differentiate into glia or NPCs, and transitional NPCs could develop into neurons of three lineages (Figure 1H,I; Figure S1B, Supporting Information).

Collectively, we generated a collaborative database that describes retinal development, laying the groundwork for subsequent analyses.

### 2.2 Construction of Gene Regulatory Networks in Retinal Progenitor Cell‐Producing Cells at the Transcriptomic and Epigenetic Levels

**Figure 2:**
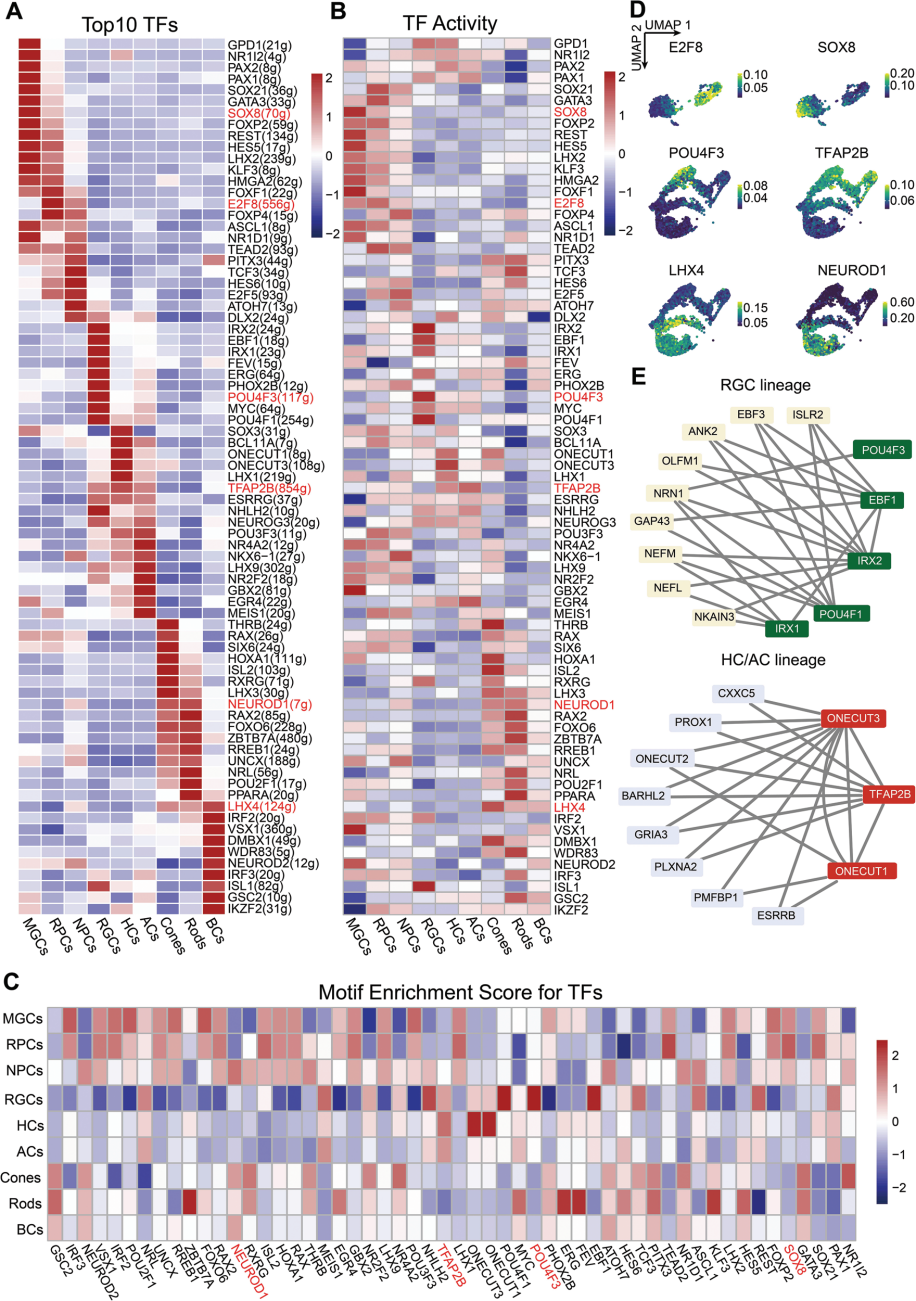
Transcriptomic and epigenetic patterns of GRNs in RPCs and their derived cells. Heatmap of A) transcriptional expression, B) gene activity, and C) their motif enrichment score of the top 10 TFs predicted by SCENIC analysis in all cell types. D) UMAP embedding showing TF regulon activity specific to several developmental lineages. E) Top GRNs of RGC and HC/AC lineages predicted by the SCENIC analysis.

To identify which TFs are crucial for the differentiation of RPCs into major retinal cells and for the maintenance of their specific cell end‐states, we performed Python implementation of a single‐cell regulatory network of inference and clustering (SCENIC) analysis. Moreover, we assessed the relative accessibility to chromatin and enrichment of motifs for these TFs (**Figure** **2**).

The results predicted a series of TFs, with highly similar transcriptomic and epigenomic levels, as vital for the fate of specific cell types (Figure 2A–C; Figure S2A, Supporting Information). UMAP embedding of SCENIC TF activity revealed four developmental branch endpoints derived from RPCs, which were demarcated and represented by respective TFs reported to affect the development of related lineages (Figure 2D), such as SOX8 for the MGC lineage, POU4F3 for RGC lineage, TFAP2B for HC/AC lineage, and LHX4 and NEUROD1 for BC/PH lineage.^[^ ^21^, ^23^, ^24^, ^25^ ^]^

Next, we screened critical cell‐type‐specific TFs at the epigenetic level from the top predicted TFs; the 10 genes regulated by at least two key TFs, according to the weight value, were selected to depict the top GRNs of related TFs for each cell linage (Figure 2E; Figure S2B,C, Supporting Information). For instance, the Iroquois homeobox protein family TFs (IRX1 and IRX2) exhibited a marked influence on genes required for RGC development, such as EBF3, NRN1, and NEFL,^[^ ^22^, ^26^ ^]^ whereas ONECUT family members had regulatory effects on HC/AC lineage‐specific factors, including PROX1 and BARHL2.^[^ ^27^, ^28^, ^29^ ^]^

In summary, we identified fate‐determining TFs for RPCs and their products, constructed corresponding top GRNs for each lineage, and demonstrated the cell‐type specificity of several important TFs at the transcriptomic and epigenomic levels.

### 2.3 Classification of Human Fetal Müller Glial Cells

**Figure 3:**
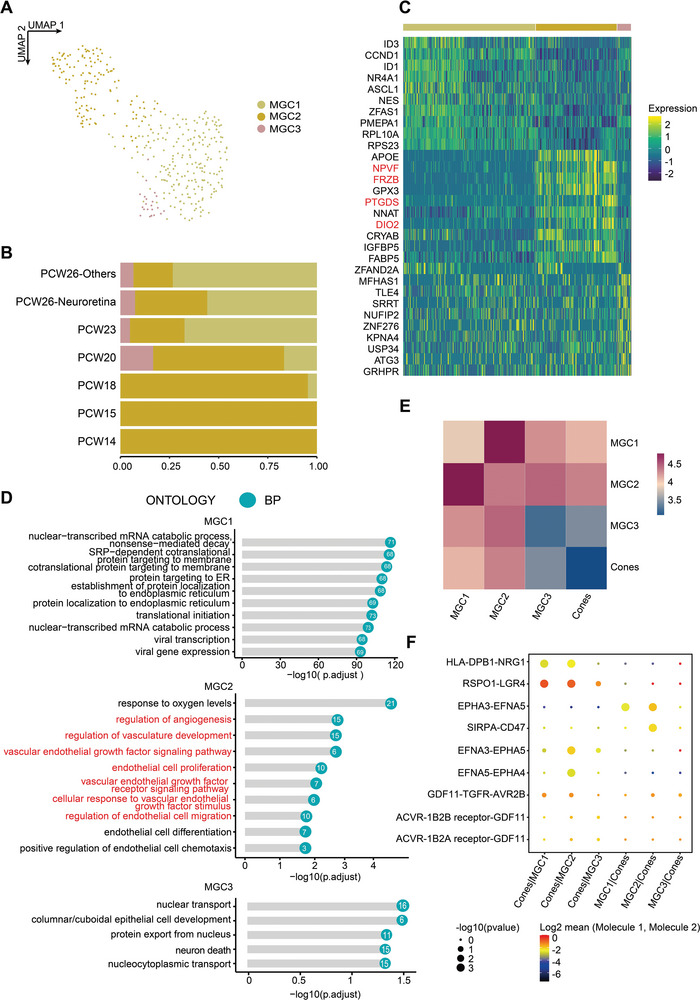
Characterization of MGC subpopulations. A) UMAP embedding of MGC subpopulations. B) Proportion of cell ages for MGC subpopulations. C) Heatmap showing the top 10 DEGsof MGC subclusters. D) Bar plots showing the top Gene Ontology: Biological Progress (GO:BP)terms calculated by using DEGs in MGC subpopulations. E) Heatmap showing the interaction relationship between MGC subpopulations and cones. F) Dot plots showing the ligand‐receptor interaction between MGC subpoplations and cones.

Next, we took a closer look at how retinal glial cells develop. Since astrocytes were not annotated in our data, we classified MGCs and identified a subset of MGCs located in the macular. According to their distinct features, MGCs were divided into three subsets (**Figure** **3**A–C). Gene ontology analysis of their respective differentially expressed genes (DEGs) revealed that MGC1 was primarily enriched in protein‐related biological processes, whereas MGC2 functioned in response to the external environment, especially hypoxia, indicating that these cells might participate in retinal angiogenesis during embryonic retinal development (Figure 3D). Notably, MGC2 highly expressed a set of genes that are reportedly specifically expressed in macular retina,^[^ ^16^ ^]^ including NPVF, FRZB, PTGDS, and DIO2, suggesting that MGC2 might represent the predominant subgroup of MGCs localized in the macular retina. We also observed highly specific expression of FABP5 and IGFBP5 in the MGC2 subset, which might represent novel markers for macular MGCs. In addition, the MGC2 subcluster manifested a stronger interaction with cones—the only macular photoreceptor cell type—further supporting the notion that the MGC2 subset represents macular MGCs (Figure 3E). Regarding their ligand‐receptor interactions, MGC2 and cones interacted during embryonic development (Figure 3F), particularly during the growth and developmentalstages, including RSPO1‐LGR4 involved in the WNT pathway and EFNA3‐EPHA5 implicated in mediating developmental events and response to hypoxia. Collectively, these findings suggest that embryonic MGC2 might play a vital role in the normal development of embryonic macular neurons and function as the key cell type responsible for macular retinal injury response under pathological conditions.

In summary, we characterized the transcriptomic characteristics and functions of embryonic MGC subgroups and found that MGC2 might represent the unique macular cluster.

### 2.4 Differentiation of Human Fetal Müller Glial Cells

RPCs develop in two directions; MGCs arise from late RPCs.^[^ ^14^, ^16^ ^]^ Although each type of retinal neuron has its own precursor cells, few reports have discussed immature MGCs or MGC precursors in the embryonic retina. To detect MGC precursors, we classified RPCs into subpopulations and analyzed their respective features and functions. We then assessed their internal discrepancies and potential to differentiate into MGCs (Figure S3A,B, Supporting Information). RPC9 was excluded from subsequent analysis as it was considered a redundant cluster due to its low quantity (27 cells, 0.135% of the total RPC cell count) and its presence in the non‐retinal sample (Table S5, Supporting Information).

**Figure 4:**
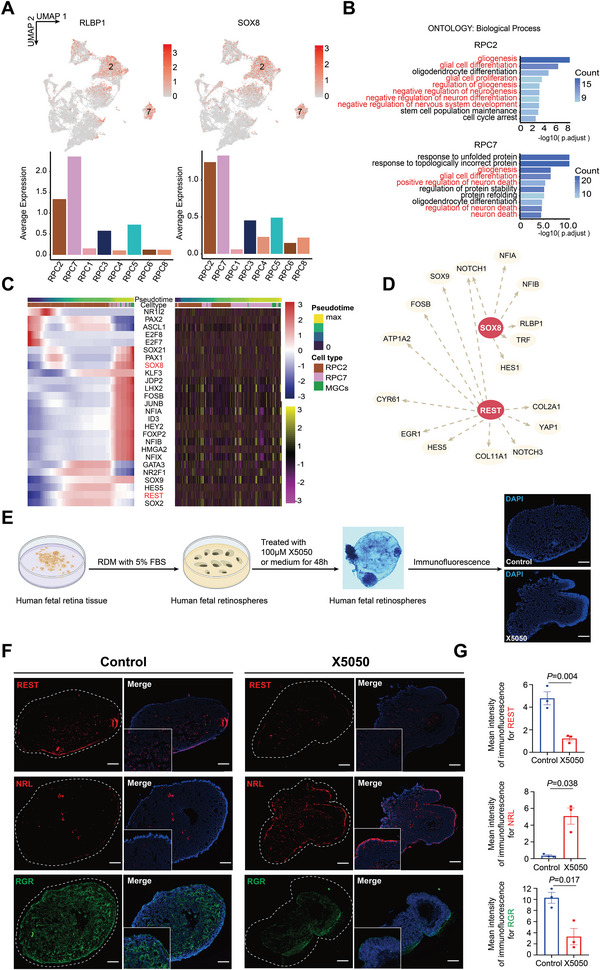
Confirmation of a MGC precursor subset and validation of the role of REST in MGC development. A) UMAP embedding and histograms showing expression of RLBP1 and SOX8 in RPC subpopulations. B) The top GO:BPterms calculated using DEGs in RPC2 and RPC7. C) Heatmap of the expression and chromVAR activity scores of the top variable motifs for TFs found in the MGCs over pseudo‐time (bar at the top) and cell type (bar at the top). D) SOX8 and REST regulatory networks predicted by SCENIC analysis. E) Schematic of the experimental design for REST inhibition in retinospheres. F) Immunofluorescent staining of REST, NRL, and RGR in the control and X5050‐treated fetal retinospheres after 48 h. Scale bar, 100 µm. G) Bar plots showing the mean immunofluorescence intensity for REST, NRL, and RGR staining. Data are presented as mean ± SEM. *n* = 3 independent replicates for each retinosphere. Student's *t*‐test was performed between each X5050‐treated retinosphere and the control for each gene. *p*‐values between all retinospheres and control are provided.

Owing to their emergence at an early stage (PCW9 and PCW10) with high cycling and enriched function related to mitosis (Figures S3C,D and S4A, Supporting Information), RPC1, RPC4, and RPC6 subclusters were identified as early pluripotent RPCs. Meanwhile, from PCW18 onward, RPC2 and RPC7 emerged and shared upregulation of NFIB and NFIX and downregulation of MKI67 (Figure S4B, Supporting Information), suggesting their gradual exit from the cell cycle. Additionally, several genes closely related to MGC development and maintenance, such as RLBP1 and SOX8,^[^ ^16^, ^23^, ^30^ ^]^ were highly expressed in these two subgroups. Moreover, their related functions enriched in glial cell proliferation and differentiation (**Figure** **4**A,B), suggesting that these cells were likely undergoing MGC differentiation. Therefore, we speculated that RPC2 and RPC7 represented the MGC precursor populations.

To confirm this hypothesis and investigate the MGC developmental process, we performed pseudo‐time analysis and constructed a map of the relative expression of crucial TFs and corresponding enrichment of their motifs at progressively differentiated states (Figure S5A, Supporting Information; Figure 4C). Consistent with our hypothesis, RPC2 and RPC7 subgroups were widely observed at the beginning of the developmental track where MGCs appeared. Furthermore, RPC2 and RPC7 exhibited upregulated expression of CXCR4, SPP1, and transferrin (TRF) compared to the other RPC subgroups (Figure S5B, Supporting Information), all of which are associated with MGC development.^[^ ^31^, ^32^, ^33^, ^34^, ^35^, ^36^ ^]^ Additionally, REST, which was predicted to define the final state of RPCs and MGCs by SCENIC analysis, exhibited high chromatin accessibility and expression in RPC2 and RPC7, whereas SOX8 showed a gradual increase in chromatin accessibility and expression from RPC7 to MGCs. Hence, these genes likely have different roles in the emergence and maintenance of MGCs derived from glial cell precursors among RPCs at both transcriptional and epigenetic levels.

We then sought to further explore the roles of SOX8 and REST in the development of MGCs from RPCs and found that a series of MGC‐related genes, including TRF, RLBP1, NOTCH1, and SOX9, were predicted to be regulated by these two factors (Figure 4D). TRF was the most strongly influenced by SOX8, with a specific transcriptional overlap between SOX8 and TRF (Figure S5C, Supporting Information), indicating that a regulatory relationship might exist.

To verify the role of REST in the existence of embryonic RPCs and MGCs, we cultured and induced embryonic retina in the early PCWs (PCW11–PCW14) before MGC emergence to differentiate and form retinospheres, which were then treated with the REST inhibitor, X5050 (Figure 4E; Figure S6A, Supporting Information). The expression of RGR (labeled MGCs) was significantly reduced, whereas NRL (labeled rods) largely increased following X5050 treatment (Figure 4F,G). Moreover, no significant difference was observed in the expression of BC and cone markers after treatment. However, the retinal differentiation structure was more complete and ordered, with the cells uniformly arranged and exhibiting a more mature morphology in their respective corresponding retinal layers compared to that observed before treatment (Figure S6B, Supporting Information). Hence, REST might influence the generation of retinal neurons in addition to playing a prominent role in the maintenance of RPCs and MGCs.

Overall, we have described the development of MGCs from RPCs, defined a subset of MGC precursors, and demonstrated the unique role of REST in retinal development in vitro.

### 2.5 Differentiation Potential of Neurogenic Retinal Progenitor Cells and Their Intrinsic Connections

**Figure 5:**
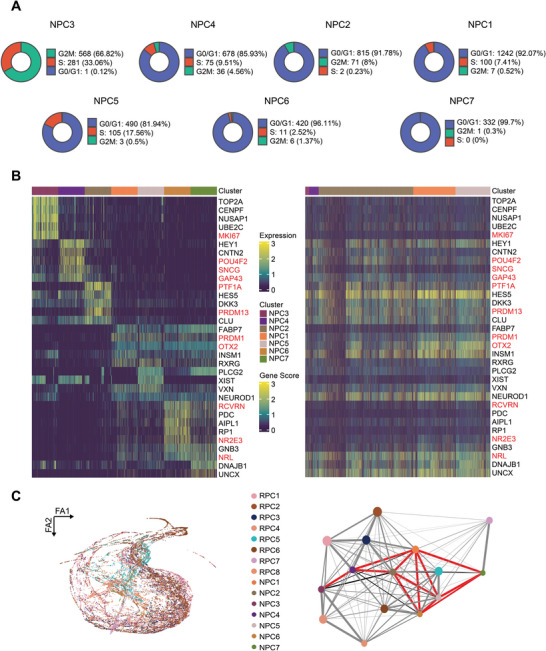
Resolution of the intrinsic relationship among NPCs and their developmental trajectories. A) Proportion of cells in the G1, S, and G2M phase in NPC subclusters. B) Heatmaps showing gene expression and gene score in NPC subclusters. C) Force‐directed graph (FDG) visualization of the differentiation trajectory of RPCs and NPCs. Partition‐based graph abstraction (PAGA) trajectory model imposed on the FDG visualization of the differentiation trajectory. The size of the dots is proportional to the number of cells in the clusters.

To gain insights into neuronal cell differentiation,^[^ ^15^, ^17^ ^]^ we first subclassified NPCs to define their identities (Table S6, Supporting Information;Figure S7A,B, Supporting Information). Cells in the NPC3 subcluster emerged the earliest and expressed high levels of stemness‐related genes (Figure S7C,D, Supporting Information). A total of 99.9% of these cells were in the G2M/S phase, indicating that NPC3 was the transitional pluripotent subpopulation developed from RPCs (**Figure** **5**A). Based on transcriptional and epigenetic levels of their respective DEGs in the other NPC subpopulations and related enriched functions, we identified NPC4, which exhibited high specific expression of POU4F2, GAP43 and SNCG, as the RGC lineage precursors, NPC2 with highly specific expression of TFAP2C, PTF1A, and PRDM13 as HC/AC lineage precursors, and the remaining NPC subclusters (NPC1, 5, 6, and 7) with highly specific expression of OTX2, RXRG, and other related genes as BC/PH lineage precursors (Figure 5B; Figures S7E and S8A, Supporting Information).

Next, we conducted a partition‐based approximate graph abstraction (PAGA) analysis on the subclusters of RPCs and NPCs (Figure 5C; Figure S8B, Supporting Information). The internal connection among the NPC subpopulations was clearer than that among the RPC subpopulations. Moreover, the connected trajectories of retinal‐specific neuronal precursors presented a gradual limited branching process, suggesting a certain level of regularity associated with the generation of retinal‐specific neural precursors. The pluripotent NPC3 subcluster was first restricted to the RGC lineage by default, after which the specification of several cells shifted toward the HC/AC lineage at a critical pseudo‐time of fate choice. Once the strong influence of the related BC/PH lineage arose, the developmental trajectory branched again.

In summary, we explored and characterized the internal heterogeneity of NPC subclusters and clarified a closely connected developmental trajectory within NPCs.

### 2.6 Chromatin Accessibility and Expression Dynamics of Human Fetal Neuronal Specification

**Figure 6:**
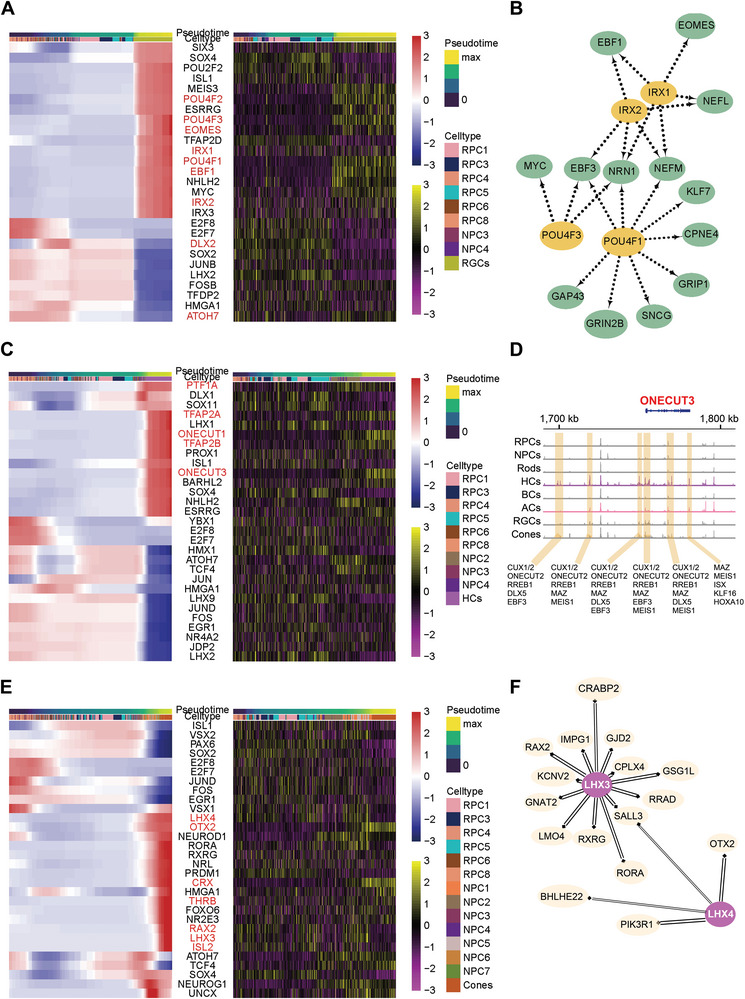
Transcriptomic and epigenetic atlas of human developing retinal neuronal lineages. A) Heatmap of the expression and chromVAR activity scores of the top variable motifs for TFs found in the RGCs over pseudo‐time (bar at the top) and cell type (bar at the top). B) The regulatory networks of key TFs in the RGC lineage predicted by SCENIC analysis. C) Heatmap of the expression and chromVAR activity scores of the top variable motifs for TFs found in the HCs over pseudo‐time (bar at the top) and cell type (bar at the top). D) Coverage plot of accessibility near ONECUT3 split by cell type. Gene color represents the direction of gene translation (red: right). E) Heatmap of the expression and chromVAR activity scores of the top variable motifs for TFs found in the cones over pseudo‐time (bar at the top) and cell type (bar at the top). F) The regulatory networks of LHX3 and LHX4 in the BC/PH lineage predicted by SCENIC analysis.

Combined with the results from SCENIC analysis, we further explored which TFs were responsible for the differentiation of specific neuronal lineages and constructed the combined transcriptional and epigenetic landscapes for the development of each lineage (**Figure** **6**).

The formation of RGCs, the first‐born neurons, went through multipotent‐NPC3 and committed precursor‐NPC4 transition stages (Figure 6A). The expression and accessible regions of the ATOH7 and DLX2 motifs, which are crucial for driving RGC fate restriction, were transiently abundant in the NPC4 subcluster. Meanwhile, a series of RGC‐fate‐determining TFs, including POU4F1, POU4F3, and EBF1, exhibited high enrichment at the epigenetic and transcriptional levels as development progressed from RPCs to RGCs (Figures 2 and 6A). Notably, IRX1 and IRX2 manifested a certain restriction on RGC fate specification. Thus, we examined their GRNs and found a set of genes that overlapped with factors regulated by the POU family TFs, such as EBF1, EBF3, NEFL, and NEFM, which are indispensable for developing RGCs (Figure 6B).

ATOH7^+^ NPCs generally adopt the RGC fate by default, whereas HCs and ACs arise when the FONX4/ROR*β*1‐PTF1A transcriptional regulatory cascade initiates, and negatively regulatesATOH7 expression.^[^ ^21^ ^]^ We also observed an increase in accessible regions and expression of PTF1A in NPC2 at a critical pseudo‐time; the chromatin accessibility and expression of TFAP‐2 members (TFAP2A and TFAP2B, two major downstream effectors of PTF1A) were enriched at the end stage of pseudo‐time trajectories (Figure 6C; Figure S9A, Supporting Information). Furthermore, a restrictive effect of ONECUT1 was detected on HC fate specification, as reflected by high epigenetic and transcriptional levels of ONECUT1 as well as HC‐specific factors regulated by it, such as PROX1 and LHX1. Notably, ONECUT3 demonstrated an overt effect on HC development; therefore, we subclassified HCs and detected potential cis‐regulatory elements near or within ONECUT3 to determine its role in HC specification (Figure 6D; Figure S9B,C, Supporting Information). Several variable accessibility regions were detected that were specifically open in HCs. Most of these regions became accessible in NPC2, the HC/AC precursor stage, and notably increased in HC subpopulations. We then analyzed the TF motifs in these accessible regions to determine which might activate these putative enhancers. Nearly all of these regions contained motifs for ONECUT family TFs, in particular ONECUT2. These regions may also be activated by genes that are “upstream” in the developmental trajectory, such as EBF3, MSX1, and MSX2, or may be regulated by ONECUT3 in an autoregulatory manner following its expression onset.

OTX2 and CRX—key TFs that provide differentiation potential to the BC/PH lineage—had highly accessible regions and exhibited upregulated expression during the transition phase in BC/PH precursors and were continuously expressed in BCs and photoreceptors (Figure 6E; Figure S9D,E, Supporting Information). PRDM1 and VSX2*—*two antagonistic TFs involved in BC/PH lineage specification—are vital for the fate selection of photoreceptors and BCs in the final state, respectively.^[^ ^37^ ^]^ Herein, we predicted that many TFs, besides OTX2 and CRX, were able modulate PRDM1 transcription, including THRB, RXRG, and RAX, which could maintain its expression in photoreceptor precursors and photoreceptors (Figure S9F, Supporting Information). Furthermore, key factors for BC formation, such as VSX2, VSX1, and ISL1, exhibited an increase in TF motifs and expression accompanied by a decrease in PRDM1 expression. Additionally, we observed a transient increase in ISL1 accessibility and expression in BC/PH precursors, followed by abundantly accessible motifs and increased VSX2 expression in BCs. Interestingly, LHX family TFs (LHX3 and LHX4) made discrepant contributions to BC/PH lineage specification, with LHX4, not LHX3, exhibiting a considerable regulatory effect on BC‐specific BHLHE22 and VSX1 (Figure 6F), which was consistent with the maturity regulation of Lhx4 and Isl1 on BC subtypes in mice.^[^ ^23^ ^]^

In conclusion, we demonstrated the lineage‐defining TF cascades that develop from RPCs and differentiate into specific neuronal types.

### 2.7 Maps of Embryonic Immune Cells and Human Ocular Disease‐Associated Genes

**Figure 7:**
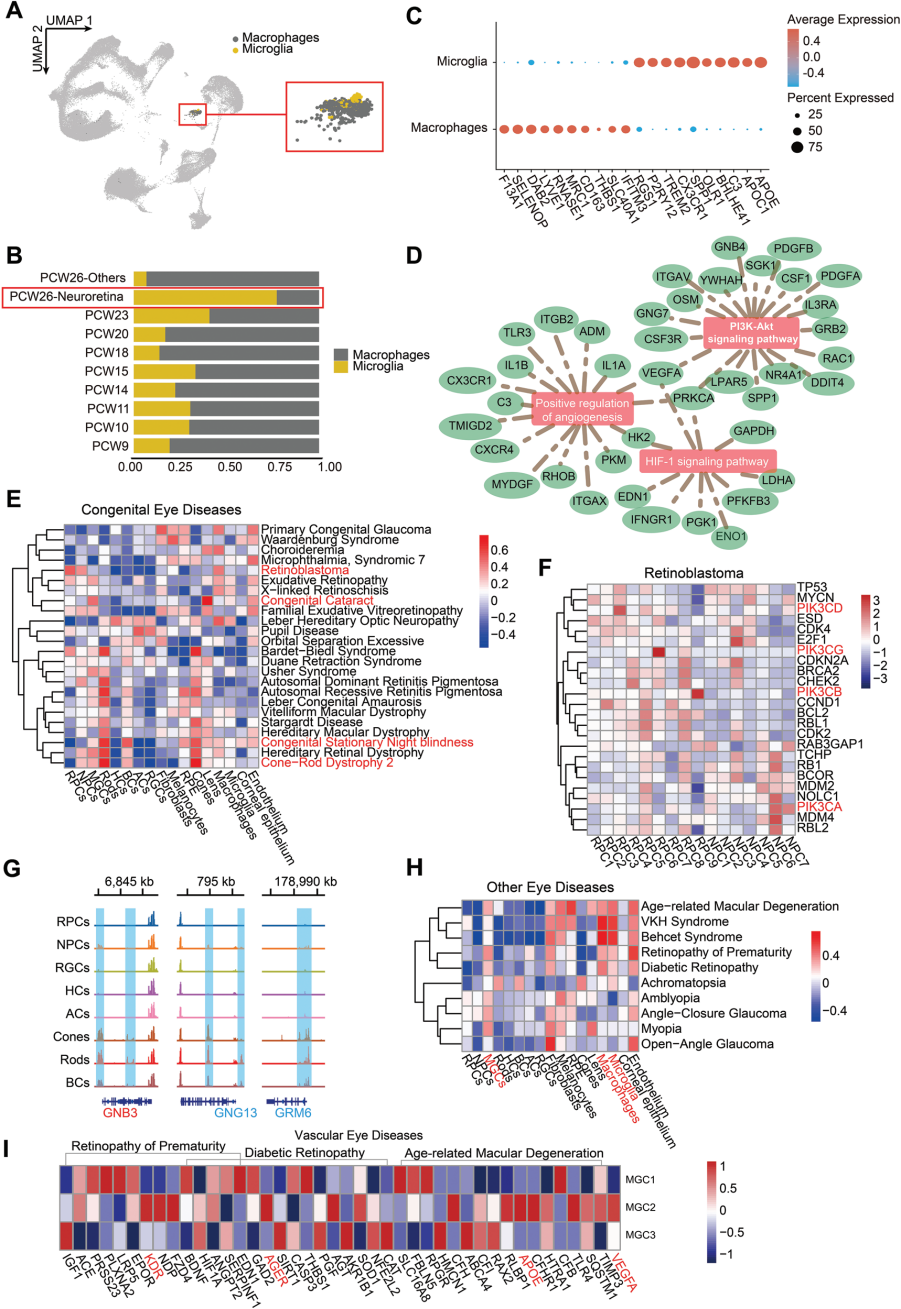
Maps reveal human fetal retinal immune cells and ocular disease‐associated genes. A) UMAP embedding showing the location of macrophages and microglia. Red box present enlarged image. B) Relative proportions of macrophages and microglia at each age. Red box highlights quantity differences. C) Dot plot showing the top 10 DEGs between microglia and macrophages in embryonic human eyes. D) Networks showing the GO: BP terms related to angiogenesis calculated using the DEGs in microglia. E) Heatmap showing the relative expression of congenital eye disease‐related genes in different cell types. F) Heatmap showing the relative expression of retinoblastoma pathogenic genes in RPC and NPC subtypes. G) Coverage plot showing the chromVAR accessibility of GluR6. Gene color represents the direction of gene translation (red, right; blue, left). H) Heatmap showing the relative expression of concerned eye disease‐related genes in different cell types. I) Heatmap showing the relative expression of vascular eye disease‐related genes in MGC subclusters.

Given that microglia and macrophages are essential for normal retinal development, we annotated these cell types by their respective specific markers and found a quantitative dominance of microglia in the retinal region (**Figure** **7**A,B). We further evaluated their discrepancies in the developing eyes and observed a set of DEGs that might serve as their biomarkers in human embryonic eyes (Figure 7C). Notably, microglia, not macrophages, were predicted to have a vital role in fetal eye angiogenesis, owing to the observed microglial function enrichment within the positive regulation of angiogenesis and vasculature development (Figure 7D).

Subsequently, we investigated the top 25 disease‐related genes of various congenital and other eye diseases in the DisGeNet (https://www.disgenet.org/) and RetNet (https://sph.uth.edu/retnet/) databases. Gene set variation analysis was then employed to explore the distribution of these genes. Several characteristic gene sets in genetic eye diseases with definite morbigenous deficits were highly expressed in specific cell types (Figure 7E), such as photoreceptor defects in cone‐rod dystrophy 2 and lens degeneration in congenital cataracts.

Retinoblastoma is a malignant tumor originating from photoreceptor precursor cells, in which disease‐related genes are enriched in RPC and NPC subclusters (Figure 7E,F). We observed elevated expression of PI3K family genes in retinal neuron precursors, including PKI3CA, PKI3CB, PKI3CD, and PKI3CG, which was consistent with the effectiveness of PI3K‐targeted inhibitors on retinoblastoma.^[^ ^38^ ^]^ In addition, congenital stationary night blindness (CSNB) is characterized by defects in BCs that affect the ON response of targeted photoreceptors; we observed high chromatin accessibility in the mGluR6 cascade members, including GRM6, GNB3, and GNG13 (Figure 7G), the mutations of which represent the third most common cause of complete CSNB.^[^ ^39^ ^]^

In other eye diseases, gene sets for age‐related macular degeneration (AMD), diabetic retinopathy (DR), and common types of uveitis were markedly more expressed in resident ocular immune cells (Figure 7H). Moreover, a degree of correlation was detected between most of these diseases and MGCs, particularly vascular eye diseases, which is consistent with the impact of MGCs on retinal angiogenesis under pathological conditions.^[^ ^40^ ^]^ In particular, MGC2 was most closely related to the pathogenic genes of three diseases, namely DR, AMD, and retinopathy of prematurity (ROP), as evidenced by the highly specific expression of VEGFA, KDR, APOE, and AGER, revealing the active involvement of MGC2 subpopulations in vascular eye diseases (Figure 7I).

Collectively, we have elaborated on the differences between microglia and macrophages in fetal development and revealed the distribution of multiple ocular disease‐related genes in various cells during embryonic eye development.

## 3 Discussion

Defining the complex developmental processes of organs requires a rigorous understanding of the gene regulatory systems at epigenomic and transcriptomic levels. Herein, we performed integrative scRNA‐seq and scATAC‐seq of the human fetal retina to establish the developmental dynamics of the entire retina and individual lineages during human embryogenesis. We then constructed the lineage‐fate‐determining GRNs to further investigate the fate specification of retinal cells. Possible sources of RPCs were identified in MGCs and the important role of REST in the MGC developmental trajectory was confirmed. Our study revealed three distinct developmental orientations of NPCs derived from RPCs and explored their internal connections. We further described the lineage‐determining TF cascades that guide the differentiation of RPCs into specific neuronal types. Finally, we highlighted the differences between embryonic macrophages and microglia, and identified genes associated with eye diseases in human fetal retinal cells.

MGCs are an important class of glial cells in the retina that retain stem cell‐like properties. Given their regenerative potential, a detailed understanding of MGC spatiotemporal development can aid the development of efficient treatments for a variety of human retinal diseases.^[^ ^41^, ^42^, ^43^, ^44^ ^]^ In this study, we subdivided MGCs into three subpopulations and explored their unique signatures. By comparing the characteristics of embryonic RPCs and adult MGCs, the embryonic MGC2 subgroup was identified in the human retina macula,^[^ ^16^, ^45^, ^46^, ^47^, ^48^, ^49^ ^]^ which exhibits a strong interplay with cones. Hence, MGCs were likely regionally heterogeneous, exerting different functions in different parts of the retina. In particular, we detailed MGC2 in the human embryonic retina macula and found that its interaction with cones may change when cones become diseased, at which time the MGCs in the macula may take on an antigen‐presenting role.

Although MGCs are thought to develop from late RPCs, there is a current dearth of data regarding whether aMGC precursor state exists that is similar to that of neuronal precursor cells. Recent studies using cell lines and animal models have demonstrated that retinal MGCs differentiate from a progenitor‐like state to immature glial cells and then to a mature state.^[^ ^9^, ^50^, ^51^, ^52^, ^53^ ^]^ Ning et al.^[^ ^54^ ^]^ defined the Ki67^−^ SOX9^+^ CRALBP^−^ GS^−^ cells as MGC precursors in retinal organoids, however, their existence in the human primary fetal retina has not been well established. Herein, we identified RPC2 and RPC7 as MGC precursors. The high expression of several cell cycle‐relevant genes suggested that RPC2 and RPC7 exited the cell cycle, while the increased expression of MGC marker genes suggested that they possess the potential to evolve into MGCs. We found that three genes, TRF, SPP1, and CXCR4, were specifically highly expressed in these two subsets, and their related biological processes represented the main functions of MGCs in the retina. TRF was found to provide essential help for the iron transport of MGCs.^[^ ^55^ ^]^ As a neuroprotective protein, SPP1 is likely involved in the nutritional and protective role of MGCs on neurons, in particular RGCs.^[^ ^31^ ^]^ The chemokine CXCR4 acts synergistically with VEGF in retinal vascular development and is considered a biological marker of vascular progenitor cells, suggesting that it may play an important role in MGC‐guided retinal angiogenesis in MGC precursors.^[^ ^32^, ^36^ ^]^ Hence, RPC2 and RPC7 could be regarded as MGC precursors, thus, providing an important link in the development of human primary retinal MGCs.

To further investigate the MGC developmental process, we identified main TFs, such as SOX8 and REST through SCENIC and Monocle analyses, which guided the terminal state of RPCs and MGCs. REST, which encodes cell‐cycle regulators and transcriptional repressors of neuronal genes, was found to be involved in the decision‐making for glial formation and maintenance of cell stemness due to the antagonistic relationship between MGCs and neuronal orientation in retinal development.^[^ ^56^, ^57^ ^]^ Notably, the inhibitor of REST led to a decrease in MGCs, a marked increase in rods, and a more regular arrangement of BCs and cones, indicating the formation of a more mature retina. Moreover, REST was found to induce the in vitro differentiation of RPCs to MGCs and might, therefore, represent a new target for the promotion of retinal neuron regeneration and alleviation of vision loss.

Regarding the neuronal development of the retina, retinal formation is a limitation of multiple precursor cells’ potential. Each fate decision of RPC‐generatedcells requires repetitive branching of genetic programs; neuronal precursor cells are not an exception.^[^ ^8^, ^58^, ^59^, ^60^, ^61^, ^62^, ^63^ ^]^ However, it remains unclear how neuronal precursor cells connect and develop internally. We found that NPC subgroups formed three interconnected sections, suggesting that the cell state transition within NPCs was gradual and asynchronous. A better understanding of the dynamic internal connections formed during the development of the 7 NPC subpopulations was achieved. This could effectively identify the differentiation potential of NPCs and assist in determining the direction of MGC differentiation and stem cell reprogramming, thus promoting the progress of regeneration to replenish lost neurons, repair retina, and treat severe retinal diseases.

Six types of retinal neurons are terminal cells in the development of NPCs and undertake important roles in retinal photoelectric signal transduction. The combined effect of cis‐regulatory regions on lineage‐defining factors complicates the fate of retinal neurons. The distinct terminal fate specification of multipotent RPCs might represent how dominant phenotypes are generated via the effects of gradually restrictive TFs, suggesting that different lineage‐determining TFs cooperatively regulate neuronal specification. We extended the “transcriptional dominance” model to the development of other neuronal lineages,^[^ ^64^ ^]^ as the deficiency or inhibition of crucial TFs would adopt the fate of earlier or antagonistic neuron types. We demonstrated the roles of several vital TFs involved in gradual branching development at different stages, including IRX1, IRX2, ONECUT3, LHX3, and LHX4, that assist in fate restriction of each neuron.

It is also important to investigate the cell‐type epigenome and transcriptome atlas of the developing human retina to allow the mapping of disease‐associated genes to specific cell types. Therefore, we generated genetic maps of ocular diseases, such as retinoblastoma and CSNB, which were matched to retinal cell types during the embryonic stage. Our results revealed the active involvement of resident immune cells, including macrophages and microglia, in the development of several immune ocular diseases. However, our two‐omics data may not accurately reflect the internal environment of a single cell, resulting in potential mismatching. Therefore, intercellular heterogeneity may reduce the correlation between gene expression and chromatin accessibility. Further studies are needed to construct a precise atlas of human retinal development and elucidate the mechanisms of cell‐type diversity in the human retina. Furthermore, due to the small number of human embryonic eye samples and the limited capabilities of fluorescence microscopy, the immunofluorescence images of retinospheres may not provide sufficiently high magnification and resolution, requiring additional samples and more advanced experimental methods and equipment to confirm our analytical results.

In conclusion, this study provides a high‐resolution transcriptional and epigenetic atlas of fetal eyes that will be crucial for further exploration of embryonic retinal cells in congenital diseases and regenerative medicine.

## 4 Experimental Section

### Ethics and Human Tissue Preparation

Human fetal eyes were obtained from the First Affiliated Hospital of Chongqing Medical University, the Third Affiliated Hospital of Chongqing Medical University, and the Women and Children's Hospital of Chongqing Medical University. The collection of human embryonic and fetal ocular material was approved by the Ethics Committee of Chongqing Medical University's First Affiliated Hospital and was carried out in accordance with the approved guidelines (No. 2019‐100‐2). All donors provided signed informed consent and agreed to donate aborted fetuses to this study.

Fetal eyes, at 9–26 weeks of gestation, were dissected from fetal and embryonic terminations, collected in DMEM/F‐12 (C11330500BT, Gibco), and transferred into a sterile Petri dish. The fetal eye tissue was then sectioned into pieces, followed by digestion in 3 mL of 2 mg mL^−1^ papain (LS003119, Worthington) for ≈20 min at 37 °C. Using a 30µm MACS Smart Strainer (130‐098‐458, Miltenyi), the dissociated fetal eyeball tissue was filtered to guarantee single‐cell suspensions. Enzymatic digestion was stopped by the addition of an equal volume of 10% fetal bovine serum (FBS; 10 099, Gibco) to DMEM/F‐12. The cells were then pelleted and resuspended in 1 mL of PBS for scRNA‐seq and scATAC‐seq.

### Retinospheres

Small sections of tissue (200–800 µm) were maintained in low‐attachment plates (3471, CORNING) in retinal differentiation media (RDM) comprising DMEM (C11995500BT, Gibco) DMEM/F12 1:1, 2% B27 supplement (17504‐044, Gibco), and 1% penicillin/streptomycin with 5% FBS to preserve the fetal retina. After 4–6 weeks, retinal tissue pieces fused and formed spheres capable of enduring long‐term storage in culture. Every 2 days, the medium was replaced with fresh RDM containing 5% FBS.

### RE‐1 Silencing Transcription Inhibition of Cultured Embryonic Retinosphere

X5050 (5.06026.0001, Merck Millipore) was used to inhibit REST expression in fetal retinospheres (PCW11–PCW14). The culture media were supplemented with X5050 at a concentration of 100 µM. The same amount of RDM (3 mL) was added to the cultures for the control group. Retinospheres were collected for further processing. After the cultures were maintained with the REST inhibitor or RDM for 48 h, immunohistochemistry was performed.

### Immunofluorescence Staining of Frozen Sections

Human fetal eyes were fixed overnight in 4% paraformaldehyde. The samples were then washed in PBS, dehydrated overnight at 4 °C with 20% sucrose solution, embedded in 30% sucrose and OCT (Tissue‐Tek O. C. T. Compound) at a 1:1 ratio, and snap‐frozen in dry ice. Thin (8–10 µm) cryosections were obtained using a cryostat (Leica). For immunohistochemistry, sections were permeabilized for 20 min at 20–28 °C in 0.3% Triton X‐100 (P0096, Beyotime), washed three times in PBS, blocked for 2 h at 37 °C with 10% normal goat serum, and incubated for 16 h at 4 °C with primary antibodies diluted in prefabricated solution. Primary antibodies specific for NRL (1:200; sc‐374277, Santa Cruz), RGR (1:500; ABP56042, Abbkine), CALBINDIN (1:200; 66394‐1‐Ig, Proteintech), PKC (1:200; AF6197, Affinity), CALRETININ (1:200; 92635T, CST), BRN3A (1:200; sc‐8429, Santa Cruz), OPSIN‐Blue (1:300; AB5407, Millipore), OPSIN‐Red/Green (1:300; AB5405, Millipore), and REST (1:200; sc‐374277, Santa Cruz) were used. Subsequently, sections were washed with PBS and incubated for 2 h at room temperature between 20 and 25 °C with secondary antibodies (1:500; A0423/A0521, Beyotime). DAPI (C1006, Beyotime) staining was used to visualize the nuclei. Images were captured using a fluorescence microscope and processed using the ImageJ software.

### Single‐Cell RNA Sequencing Data Processing and Analysis

Preprocessing and sequence alignment of data readings were performed using the Cellranger (v3.1.0) pipeline with the default and suggested parameters.^[^ ^65^ ^]^ GRCh38 served as the human reference genome for alignment. After filtering noncell‐associated barcodes and counting unique molecular identifiers, Gene‐Barcode matrices were created for each individual sample. Finally, the pipeline produced the gene‐barcode matrix, including the barcoded cells and gene expression counts. R software (v3.16.1) and the Seurat standard procedure (v3.1.2) were used for quality control, dimensionality reduction, clustering, filtering, and doublet exclusion.

The gene expression matrix was normalized and scaled using the NormalizeData and ScaleData functions. The top 2000 variable genes were selected using FindVariableFeatures for PCA analysis. The batch effect among samples was removed by Harmony (v1.0) using the top 20 principal components from PCA. Cells were divided into 65 clusters using FindClusters with the resolution parameter at 2.5 and the top 20 principal components. Cell clusters were visualized using UMAP with Seurat function RunUMAP.

### Transcription Factor Regulatory Network Analysis

The TF network was constructed by pySCENIC (v0.11.0) using the scRNA expression matrix and TFs from AnimalTFDB.^[^ ^66^ ^]^ First, a regulatory network was predicted by GRNBoost2 based on the co‐expression of regulators and targets. Next, CisTarget was applied to exclude indirect targets and to search TF binding motifs. Thereafter, AUCell was used for regulon activity quantification for every cell. Cluster‐specific TF regulons were identified according to Regulon Specificity Scores (RSS) and the activities of these TF regulons were visualized in heatmaps.

### Differentially Expressed Genes Analysis

Seurat function FindMarkers based on the Wilcoxon rank sum test was used to identify DEGs. The genes expressed in more than 10% of the cells in both groups, with an average log (Fold Change) value > 0.25 were selected as DEGs. Using the Bonferroni Correction, an adjusted *p‐*value was calculated, and a threshold of 0.05 was applied to determine if the result was statistically significant.

### Cell Type Annotation and Subtyping of Major Cell Types

The expression of canonical markers in the DEGs of each cluster was used to identify the cell type of each cluster with reference to the literature and database SynEcoSysTM (Singleron Biotechnology). Seurat Vlnplot function was used to display the expression of markers for each cell type with violin plots.

To obtain a high‐resolution map of RPCs‐producing cells, cells from the specific cluster were extracted and reclustered for more detailed analysis, following the same procedures described above, and the appropriate clustering resolution was set.

### Cell Cycle Analysis

The cell cycle score of each cell was calculated using the CellCycleScoring function implemented in the Seurat (v3.1.2) package.

### Pathway Enrichment Analysis

Gene Ontology (GO) and Kyoto Encyclopedia of Genes and Genomes (KEGG) analyses were utilized in conjunction with the “clusterProfiler” R package v3.16.1 to examine the potential functions of important clusters. Cellular component (CC), biological process (BP), and molecular function (MF) categories from the gene ontology were employed. Pathways that had an adjusted *p*‐value < 0.05 were considered to be significantly enriched. Selected significant pathways were plotted as bar plots. The average gene expression of each cell type was used as input data for the GSVA pathway enrichment analysis.

### Pseudo‐Time Trajectory Analysis

The differentiation trajectory of RPCs and their producing cells were reconstructed with Monocle3. Cells were arranged according to their degree of spatial‐temporal differentiation using DEGs. UMAP was used to perform praph_tset and dimension‐reduction and recognition trajectory was performed using the learn_graph function. Finally, the trajectory was visualized using the plot_cells function.

Palantir was applied to infer the development trajectories from RPCs to NPCs and MGCs. The raw data slot of the Seurat object was read into Palantir (v.1.0.0), and pre‐processed by Palantir including principal component analysis, diffusion maps analysis, and MAGIC imputation. The start cell was specified before running Palantir, and the terminal states were automatically determined. After that, the pseudotime and branch probabilities were calculated by Palantir.

Partition‐based graph abstraction (PAGA) was applied to infer the development trajectories of RPCs and NPCs. The draw_graph function from Python packages was used to compute the PAGA graph and Force‐Atlas2 (FA). Gene changes were reconstructed along PAGA paths for a given set of genes using the paga_path function.

### Cell–Cell Interaction Analysis

Cell–cell interactions among cells of interest were estimated according to known ligand–receptor pairs from Cellphone DB (v2.1.0) version.^[^ ^67^ ^]^ The null distribution of the average ligand‐receptor pair expression in randomized cell identities was calculated using a permutation number of 1000. A cutoff based on the average log gene expression distribution for all genes across each cell type was used to threshold the expression of each individual ligand or receptor. Heatmap_plot and dot_plot functions in CellphoneDB were used to display predicted interaction pairs that were deemed significant by having a *p*‐value less than 0.05 and an average log expression more than 0.1.

### Single‐Cell Assay for Transposase‐Accessible Chromatin Sequencing Data Processing and Analysis

The dual ended sequencing mode of the Illumina sequencing platform was used to conduct high‐throughput sequencing on samples, and FastQC software was used to conduct quality control analysis on the preprocessed data. Low‐quality cells with < 1000 sequencing fragments or TSS enrichment < 4 were filtered out. Bin region overlaps with ENCODE Blacklist regions were excluded from downstream analysis. The iterative latent semantic indexing approach^[^ ^68^ ^]^ was then used to reduce the dimension of the sparse insertion counts matrix. The canonical correlation analysis was applied to match scRNA‐seq and scATAC‐seq data. Clustering was performed using the addClusters function in ArchR (v1.0.1). UMAP was then used to visualize the data.

scRNA data were utilized as reference datasets to train the classifier and to assign a cell type to each cell in scATAC data. The Seurat function, FindTransferAnchors, was used to align data across two datasets. Finally, this integration approach assigned the gene expression data from the scRNA‐seq cell that most closely resembled each cell in the scATAC‐seq data to the scATAC‐seq cell for each cell in the scRNA‐seq data. MACS2 was used to perform peak calling based on the aggregated insertion sites from all cells of each cell type. By choosing the peak with the highest score from each group of overlapping peaks, a consensus set of uniform‐length non‐overlapping peaks, was created. ArchR and chromVAR were utilized to evaluate gene scores and TF motif activity in scATAC data.

### Peak Calling

MACS2 was used to perform peak calling based on the aggregated insertion sites from all cells of each cell type. By choosing the peak with the highest score from each group of overlapping peaks, a consensus set of uniform‐length non‐overlapping peaks was created. In brief, peaks were first ordered according to their significance. Any peak that directly overlapped with the most significant peak was removed from downstream analysis, and only the most significant peak was retained. This procedure was then carried out on each of the other summits until none remained.

### Single‐Cell Assay for Transposase‐Accessible Chromatin Sequencing Gene Score and Transcription Factor Activity Analysis

ArchR was utilized to evaluate gene scores and TF motif activity in scATAC data. The addGeneScoreMatrix function was used to calculate gene scores with gene score models implemented in ArchR. The addMotifAnnotations function was used to determine the motif presence in the peak set from the JASPAR2020 motif dataset. The addDeviationsMatrix function was then used to compute enrichment of TF activity on a per‐cell basis across all motif annotations based on chromVAR.

### Trajectory Analysis

In ArchR, trajectory analysis from scATAC data was carried out. The addTrajectory function was used to build a trajectory on cisTopic UMAP embedding. Next, the correlateTrajectories function was utilized to perform integrative analyses for the identification of positive TF regulators via integration of gene scores with motif accessibility with pseudo‐time.

### Single‐Cell RNA Sequencing and Single‐Cell Assay for Transposase‐Accessible Chromatin Sequencing Integrated Analysis

To investigate the TFs that are critical to the development of each retinal cell type, Monocle2 (v 2.10.0)^[^ ^69^ ^]^ was first utilized to reconstruct development trajectories of RPC‐producing cell types in scRNA data. The enriched TF motifs along the cells in the scATAC data were then shown after the cells were ordered in pseudo‐time.

### Statistical Analysis

Identification of DEGs between two groups and cell types was performed using the Wilcoxon rank‐sum test. The significance of differences was determined as indicated, and differences were considered statistically significant at *p*‐value < 0.05.

SPSS 26.0 (IBM, USA) was used for data analysis. All measurement data were shown as mean ± standard error of mean. Statistically significant deficiencies between groups were determined by Student's *t*‐test. Every experiment was repeated three times. Significance was set at *p*‐value < 0.05.

## Conflict of Interest

The authors declare no conflict of interest.

## Author Contributions

R.L., J.L., P.Y., and X.Y. contributed equally to this work. Conceptualization and supervision of the study: S.H. and J.Y.; Sample contribution: P.Y., X.Y., J.C., and H.Q.; Samples obtainment and experiment: R.L., C.Z., and X.W.; Methodology and data analysis: Z.X., H.L., and J.L.; Visualization: Z.Z., X.L., and J.X.; Supervision of the study: S.H. and K.H.; Writing—Original draft: R.L. and J.L.; Writing—review and editing: S.H. and P.Y.

## Supporting information

Supporting Information

## Data Availability

The data that support the findings of this study are openly available in the Gene Expression Omnibus (GEO) repository with accession number GSE228370 at https://www.ncbi.nlm.nih.gov/geo/query/acc.cgi?acc=GSE228370, reference number [70].
