# Supplementary material for: Integrative Single‐Cell Transcriptomics and Epigenomics Mapping of the Fetal Retina Developmental Dynamics
Source: Adv Sci (Weinh). 2023 Apr 5;10(16):2206623. doi: 10.1002/advs.202206623 (PMC10238188; doi:10.1002/advs.202206623)
Supplement: Supplementary file 1 — Supporting Information [file ADVS-10-2206623-s001.pdf]

ADVANCED SCIENCE
Open Access

Supporting Information

for *Adv. Sci.*, DOI 10.1002/advs.202206623

Integrative Single-Cell Transcriptomics and Epigenomics Mapping of the Fetal Retina Developmental Dynamics

*Ruonan Li, Jiangyi Liu, Ping Yi, Xianli Yang, Jun Chen, Chenyang Zhao, Xingyun Liao, Xiaotang Wang, Zongren Xu, Huiping Lu, Hongshun Li, Zhi Zhang, Xianyang Liu, Junjie Xiang, Ke Hu, Hongbo Qi, Jia Yu, Peizeng Yang and Shengping Hou**

Supplementary material

Integrative single-cell transcriptomics and epigenomics mapping of the fetal retina developmental dynamics

Ruonan Li[1,2,3,4#], Jiangyi Liu[1,2,3,4#], Ping Yi[5#], Xianli Yang[5#], Jun Chen[6], Chenyang Zhao[1,2,3,4], Xingyun Liao[1,2,7], Xiaotang Wang[1,2,3,4], Zongren Xu[1,2], Huiping Lu[1,2,3,4], Hongshun Li[1,2,3,4], Zhi Zhang[1,2,3,4], Xianyang Liu[1,2,3,4], Junjie Xiang[1,2,3,4], Ke Hu[1,2,3,4], Hongbo Qi[6,8], Jia Yu[9,10], Peizeng Yang[1,2,3,4], Shengping Hou[1,2,3,4,11]*

[1]The First Affiliated Hospital of Chongqing Medical University, Chongqing, 400016, P. R. China.

[2]Chongqing Key Laboratory of Ophthalmology, Chongqing, 400016, P. R. China.

[3]Chongqing Eye Institute, Chongqing, 400016, P. R. China.

[4]Chongqing Branch (Municipality Division) of National Clinical Research Center for Ocular Diseases, Chongqing, 400016, P. R. China.

[5]Department of Obstetrics and Gynecology, The Third Affiliated Hospital of Chongqing Medical University, Chongqing, 401120, P. R. China.

[6]Department of Obstetrics, Women and Children's Hospital of Chongqing Medical University, Chongqing, 401147, P. R. China.

[7]Department of Medical Oncology, Chongqing University Cancer Hospital, Chongqing, 400030, P. R. China.

[8]Chongqing Key Laboratory of Maternal and Fetal Medicine, The First Affiliated Hospital of Chongqing Medical University, Chongqing, 400016, P. R. China.

[9]State Key Laboratory of Medical Molecular Biology, Department of Biochemistry and Molecular Biology, Haihe Laboratory of Cell Ecosystem, Institute of Basic Medical Sciences, Chinese Academy of Medical Sciences, School of Basic Medicine, Peking Union Medical College, Beijing, 100005, P. R. China.

[10]The Key Laboratory of RNA and Hematopoietic Regulation, Chinese Academy of Medical Sciences, Beijing, 100005, P. R. China.

[11]Beijing Institute of Ophthalmology, Beijing Tongren Eye Center, Beijing Tongren Hospital, Capital Medical University, Beijing Ophthalmology & Visual Sciences Key Laboratory, Beijing, 100730, China.

*Corresponding author: Shengping Hou Ph.D, The First Affiliated Hospital of Chongqing Medical University, Chongqing 400016, China; Beijing Institute of Ophthalmology, Beijing Tongren Hospital, Capital Medical University, Beijing, 100730, China.

E-mail address: sphou828@163.com; sphou828@cqmu.edu.cn; Telephone number: 0086-10-58265941

#These authors contributed equally to this work.

Supplementary Figures

Figure S1

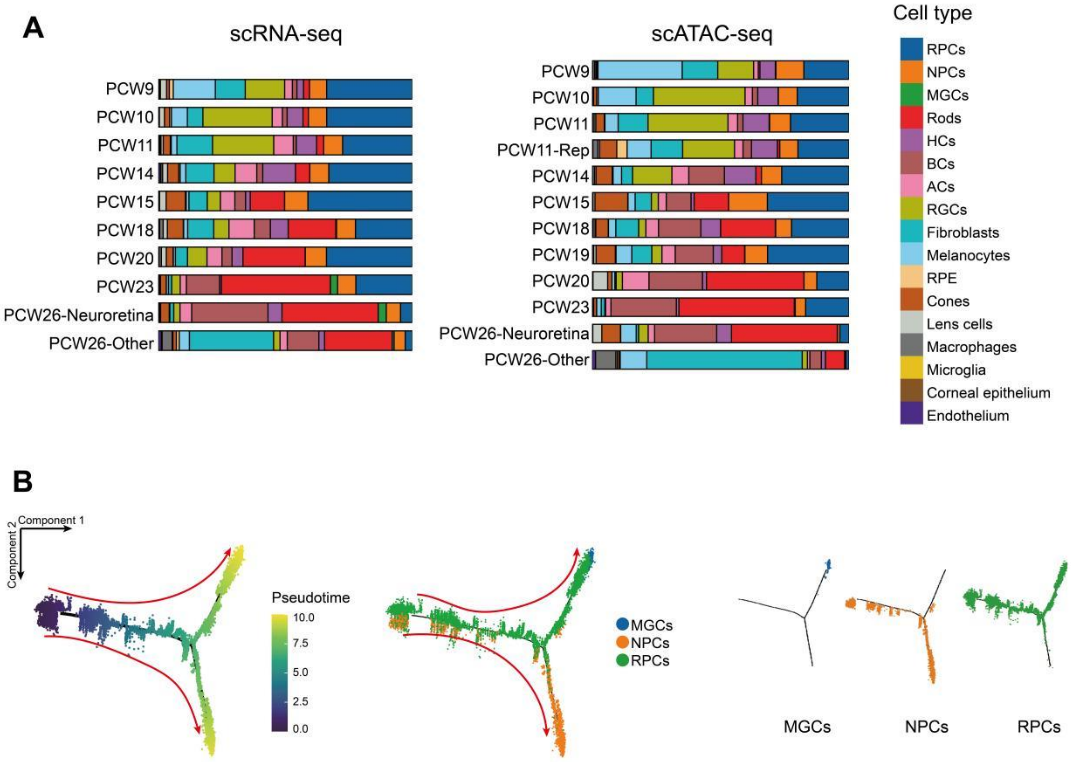

Figure S1. Proportion of ocular cell types and developmental trajectories from RPCs. (A) Proportion of cell ages for each cell type from scRNA-seq and scATAC-seq. **(B)** Plots showing the developmental trajectories from RPCs colored by pseudo-time and cell type.

Figure S2

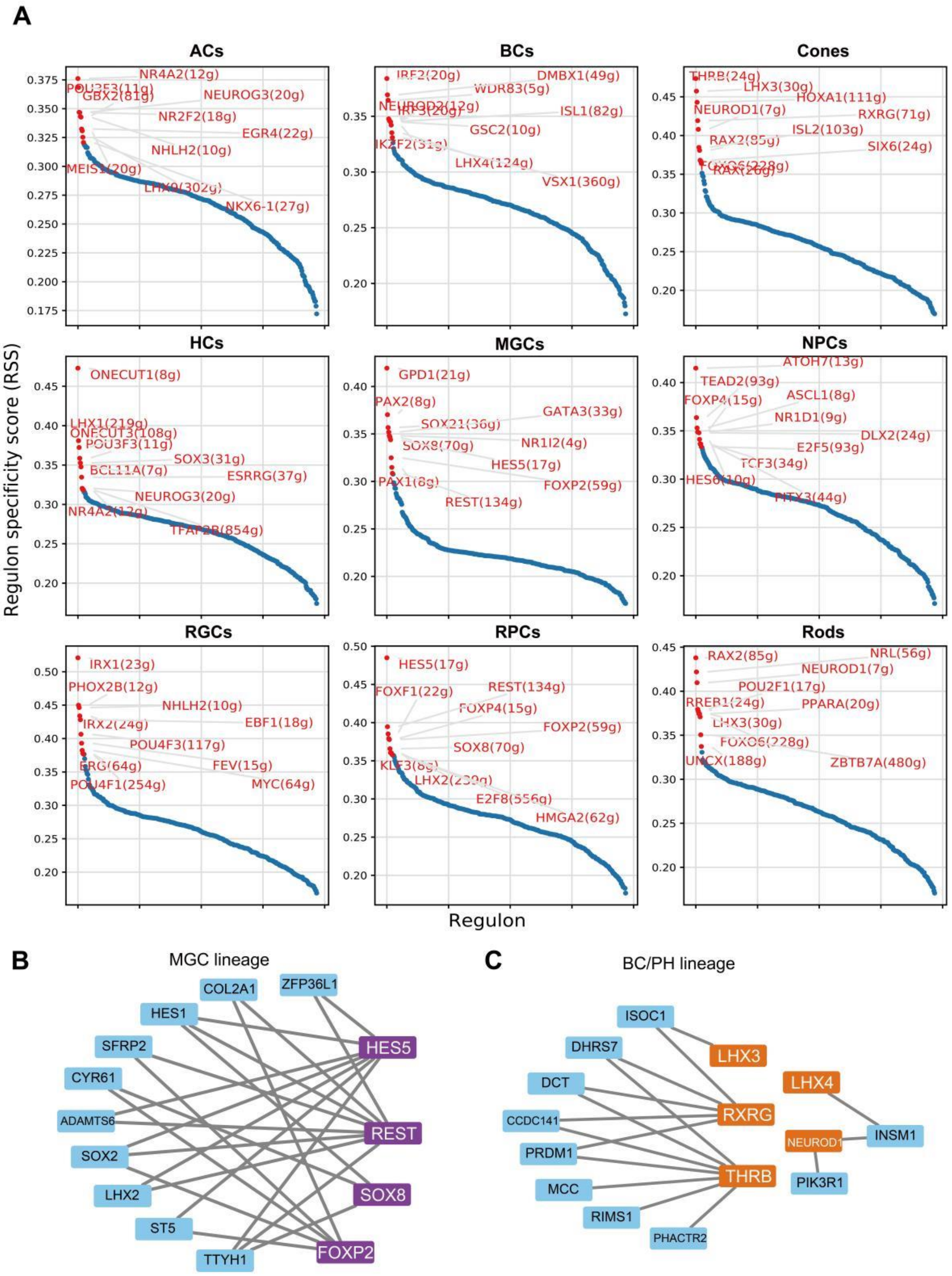

Figure S2. Top ten TFs and Top GRNs of MGCs and BC/PH lineage predicted by SCENIC analysis. (A) Plots showing the top ten TFs that determining the fate of RPCs and RPC-derived cells predicted by SCENIC analysis. **(B and C)** Top GRNs of (B) MGCs and (C) BC/PH lineage predicted by SCENIC analysis.

Figure S3

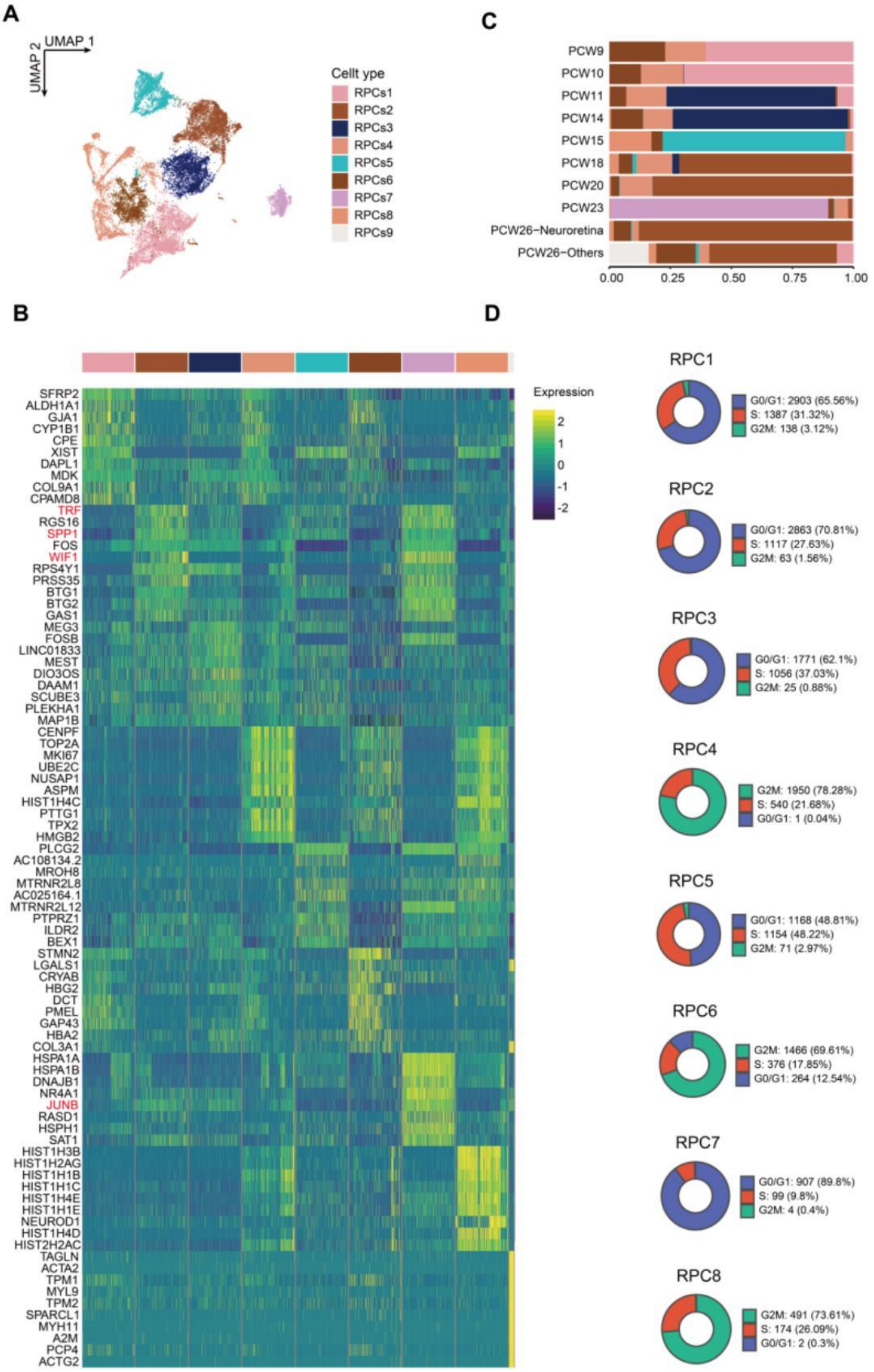

Figure S3. RPC subpopulations. (A) UMAP embedding of RPC subpopulations. **(B)** Heatmap showing the top ten DEGs of RPC subclusters. **(C)** Proportion of cell ages for RPC subpopulations. **(D)** Donut plots showing the proportion of cells in the G1, S, and G2M phase in RPC subclusters.

Figure S4

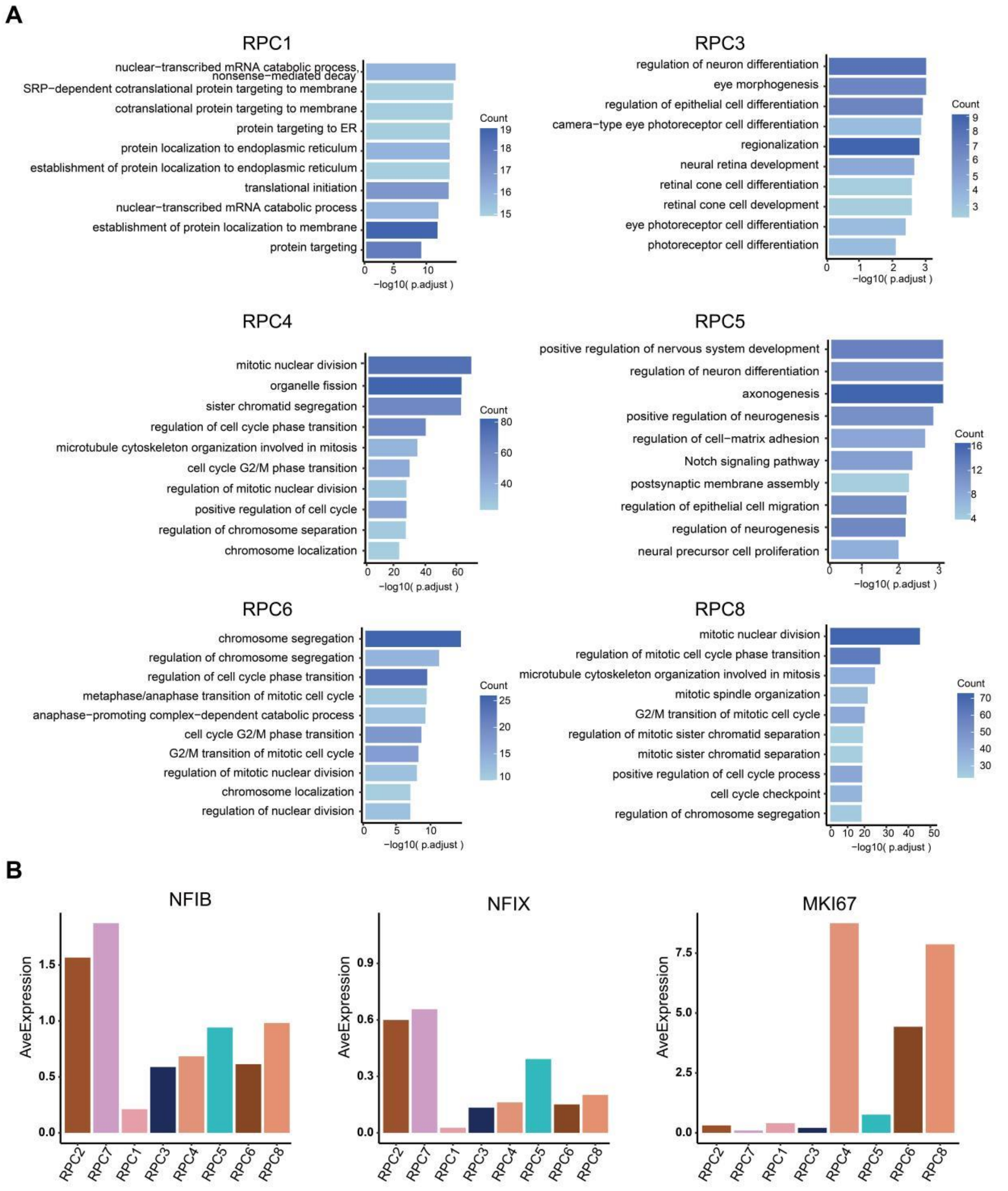

Figure S4. Features of RPC subpopulations. (A) Bar plots showing the top GO: BP terms calculated using DEGs in RPC subpopulations. **(B)** Histograms showing expression of *NFIB*, *NFIX* and *MIK67* in RPC subpopulations.

Figure S5

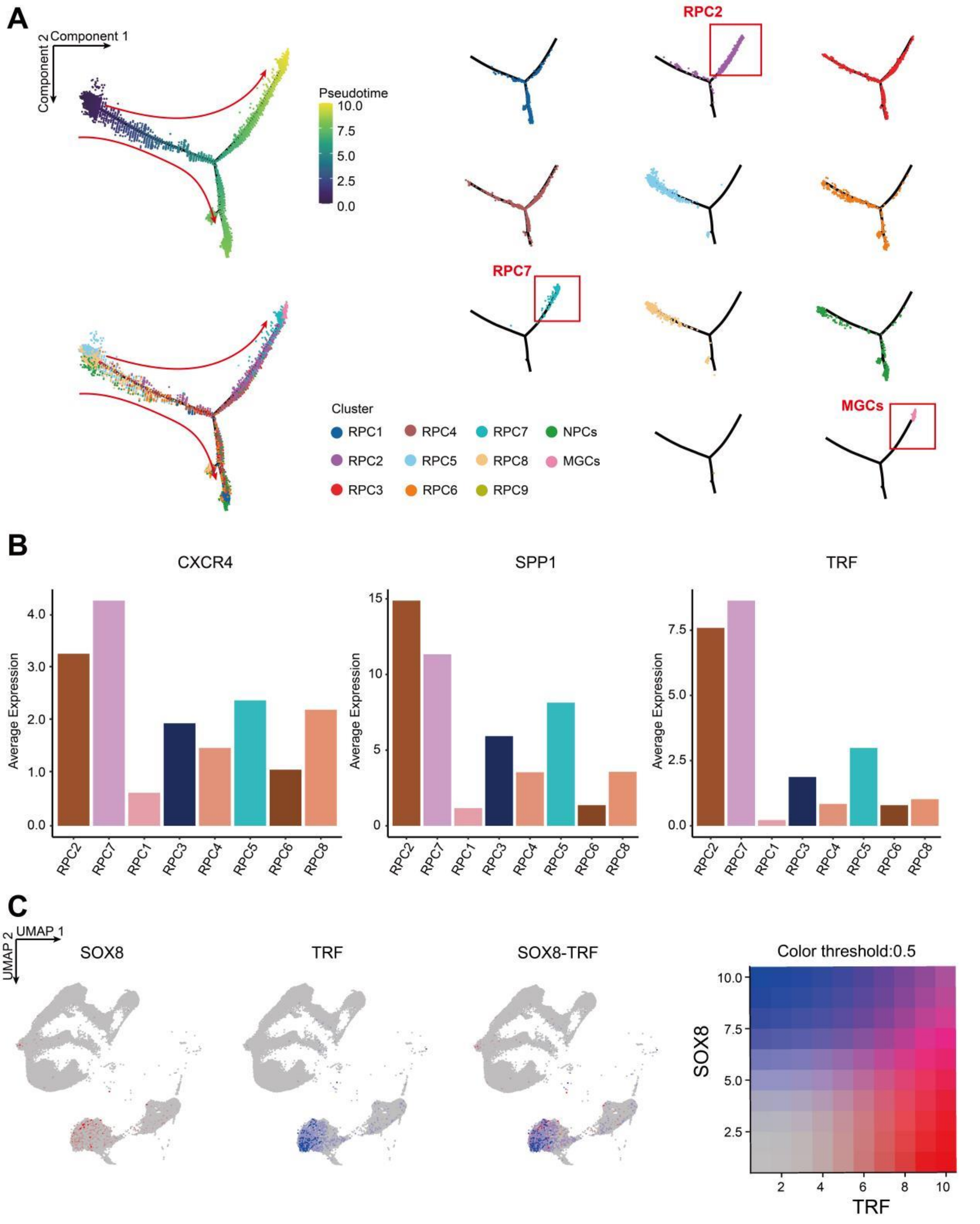

Figure S5. Development from RPCs to MGCs. (A) Plots showing the developmental trajectories from RPCs colored by pseudo-time and cell type. **(B)** Histograms showing expression of *CXCR4*, *SPP1* and *TRF* in RPC subpopulations. **(C)** The combined expression of *SOX8* and *TRF*.

Figure S6

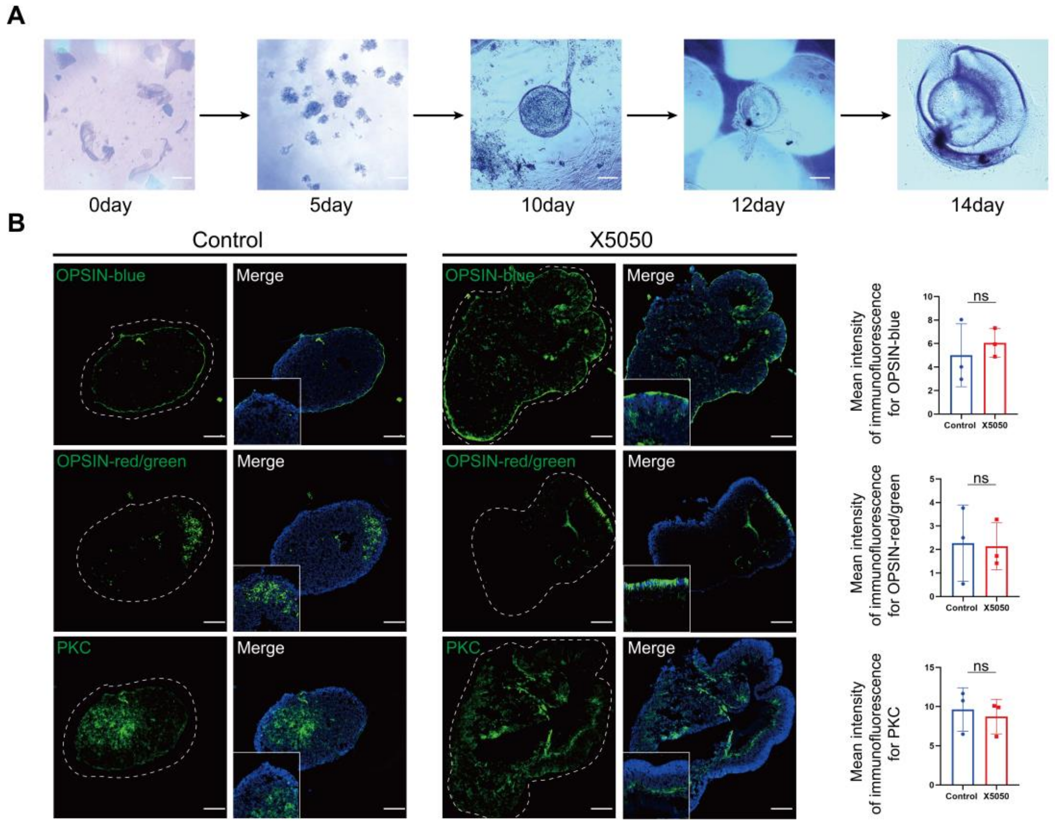

Figure S6. Validation of REST in MGC development. (A) *In vitro* retinosphere formation, Scale bar, 250 μm. **(B)** Immunofluorescent staining of OPSIN-Blue, OPSIN-Red/Green, and PKC in control and X5050-treated fetal retinospheres after 48 h. Scale bar, 100 μm. Bar plots showing the mean immunofluorescence intensity for OPSIN-Blue, OPSIN-Red/Green and PKC staining. Data are presented as mean ± SEM. n = 3 independent replicates for each retinosphere. Student's t-test was performed between each X5050-treated retinosphere and the control for each gene. p-values between all retinospheres and control are provided.

Figure S7

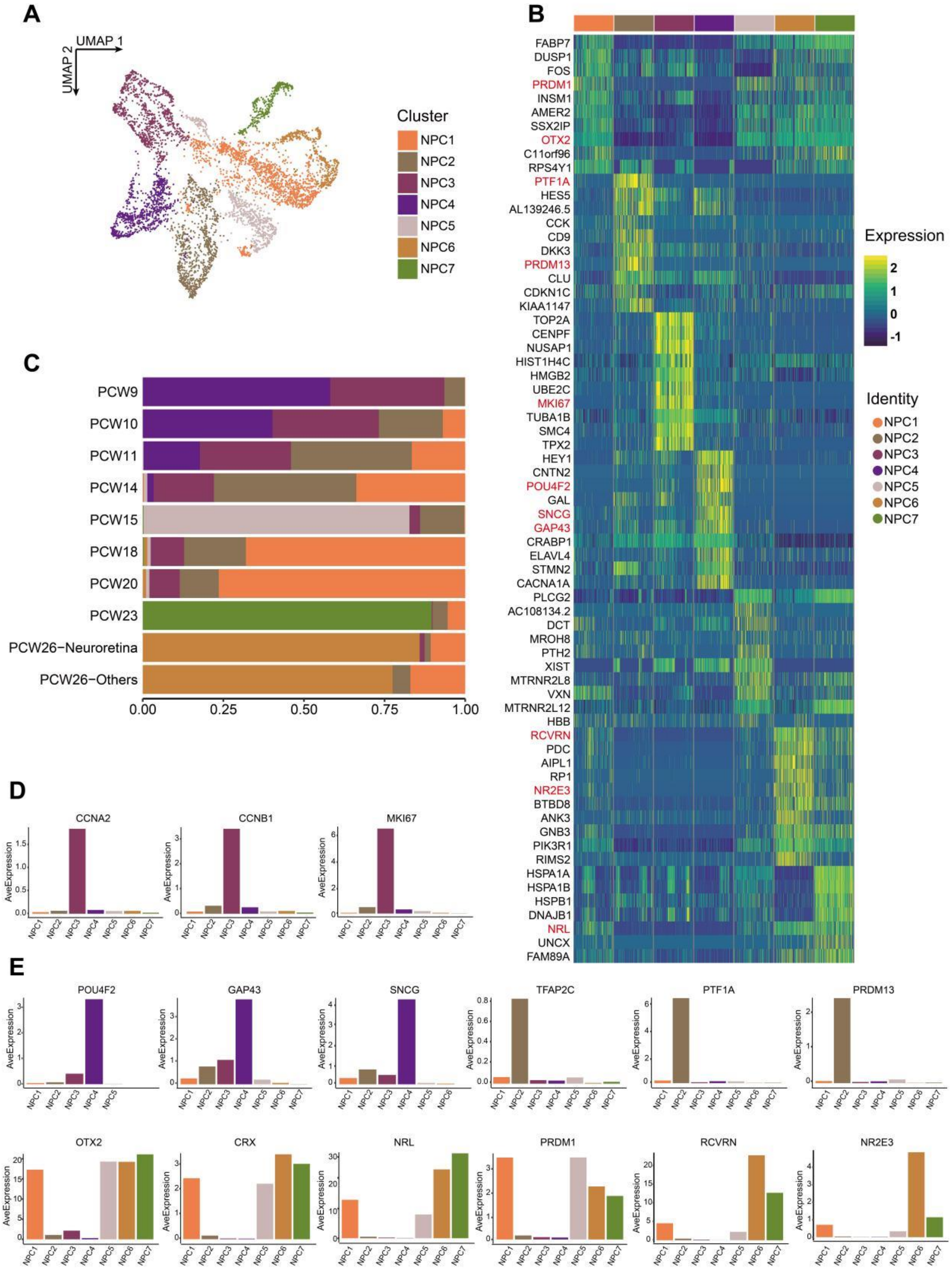

Figure S7. NPC subpopulations. (A) UMAP embedding of NPC subpopulations. **(B)** Heatmap showing the top ten DEGs of NPC subclusters. **(C)** Proportion of cell ages for NPC subpopulations. **(D)** Histograms showing expression of *CCNA2*, *CCNB1,* and *MKI67* in NPC subpopulations. **(E)** Histograms showing expression of related genes in NPC subpopulations.

Figure S8

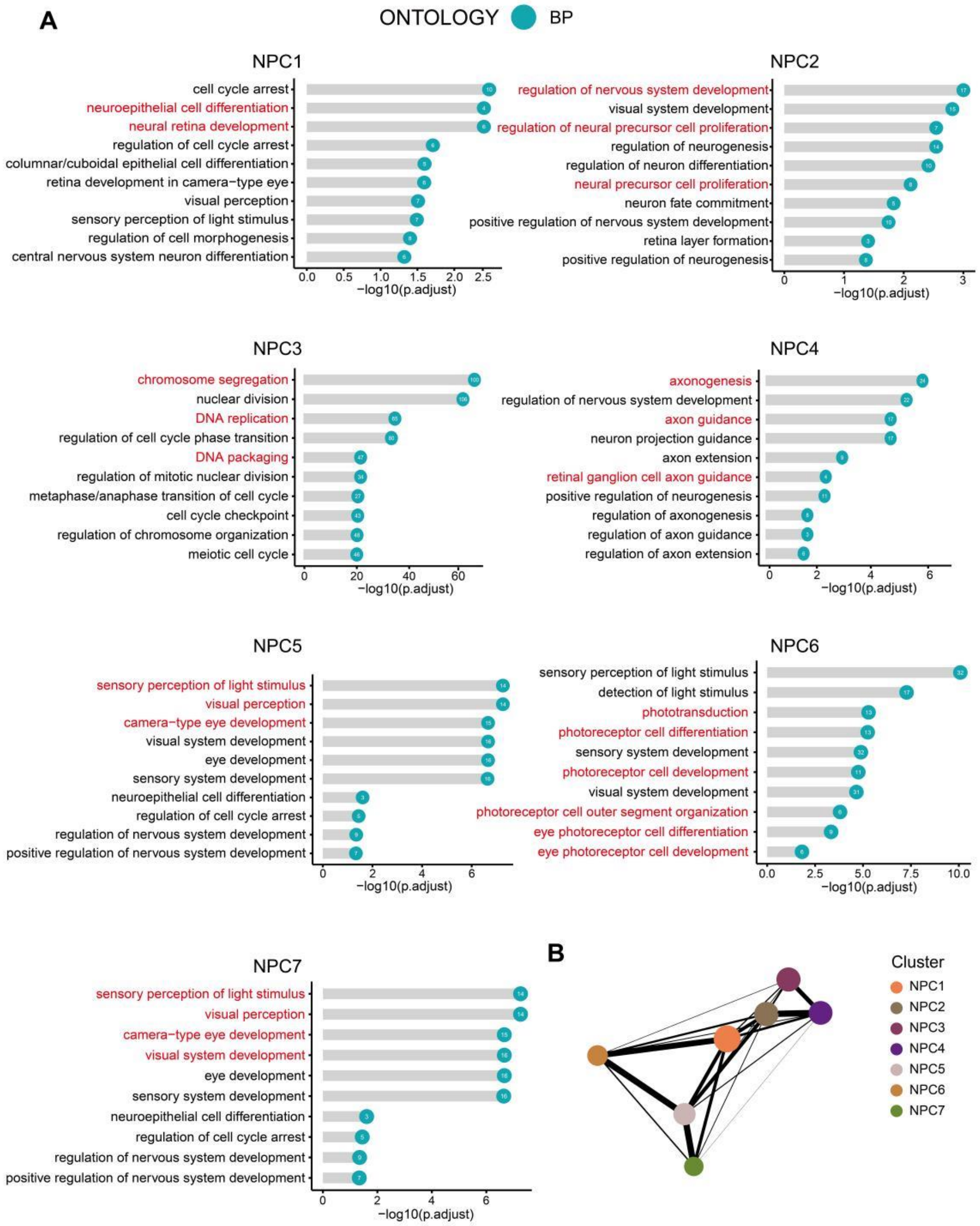

Figure S8. Features of NPC subpopulations and the differentiation trajectory within NPCs. (A) Bar plots showing the top GO: BP terms calculated using DEGs in NPC subpopulations. **(B)** Partition-based graph abstraction (PAGA) trajectory model imposed on the visualization of the differentiation trajectory in NPCs. The size of the dots is proportional to the number of cells in the clusters.

Figure S9

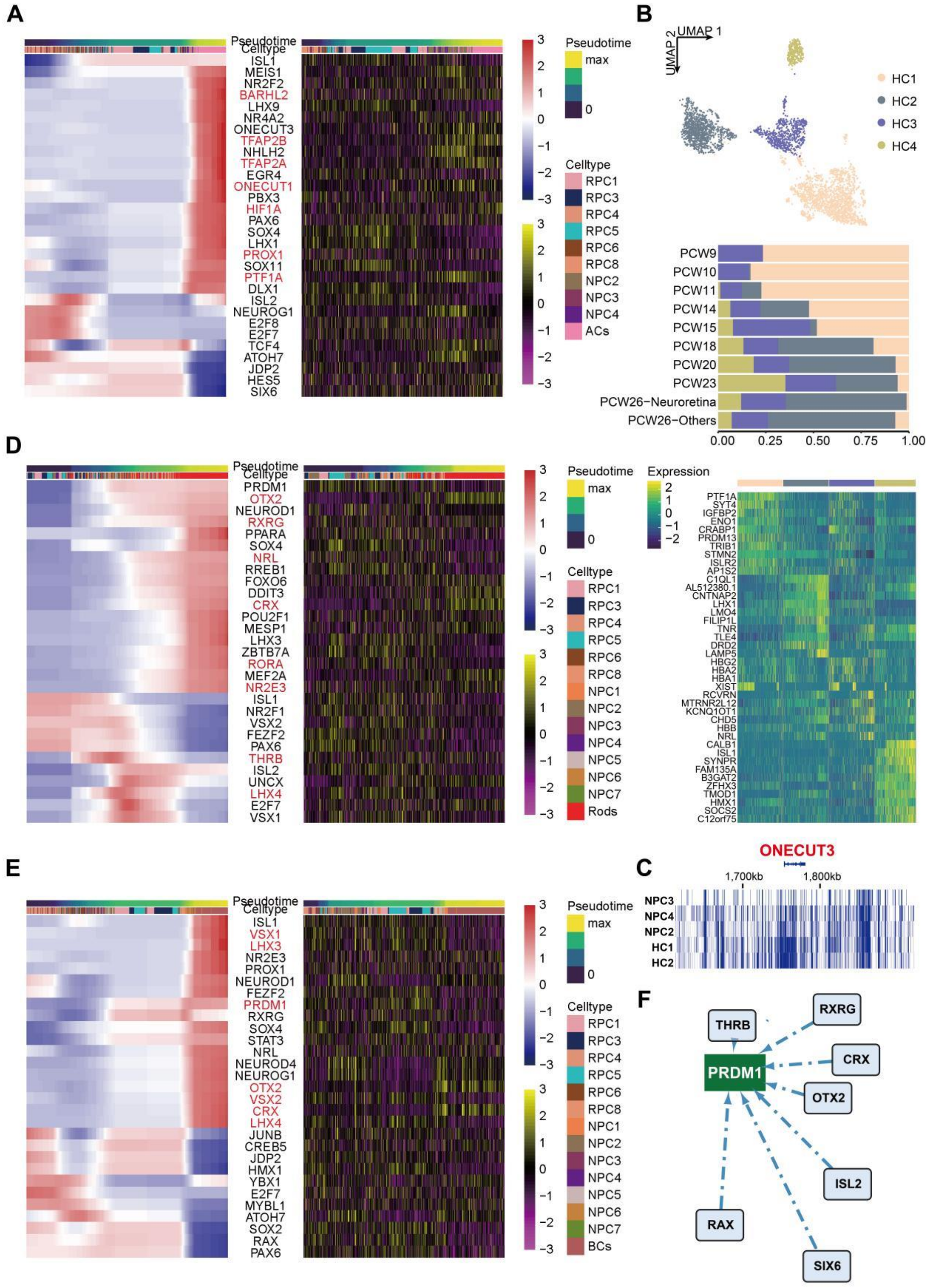

Figure S9. Transcriptomic and epigenetic atlas of human developing retinal neuronal lineages. (A) Heatmap of the expression and chromVAR activity scores of the top variable motifs for TFs found in the ACs over pseudo-time (bar at the top) and cell type (bar at the top). **(B)** UMAP embedding, proportion of cell ages and heatmap

of top DEGs for HC subpopulations. **(C)** Heatmap showing the chromVAR accessibility of *ONECUT3* in specific subclusters. **(D** and **E)** Heatmap of the expression and chromVAR activity scores of the top variable motifs for TFs found in (D) rods and (E) BCs over pseudo-time (bar at the top) and cell type (bar at the top). (**F**) The regulatory networks of *PRDM1* predicted by the SCENIC analysis.

Figure S10

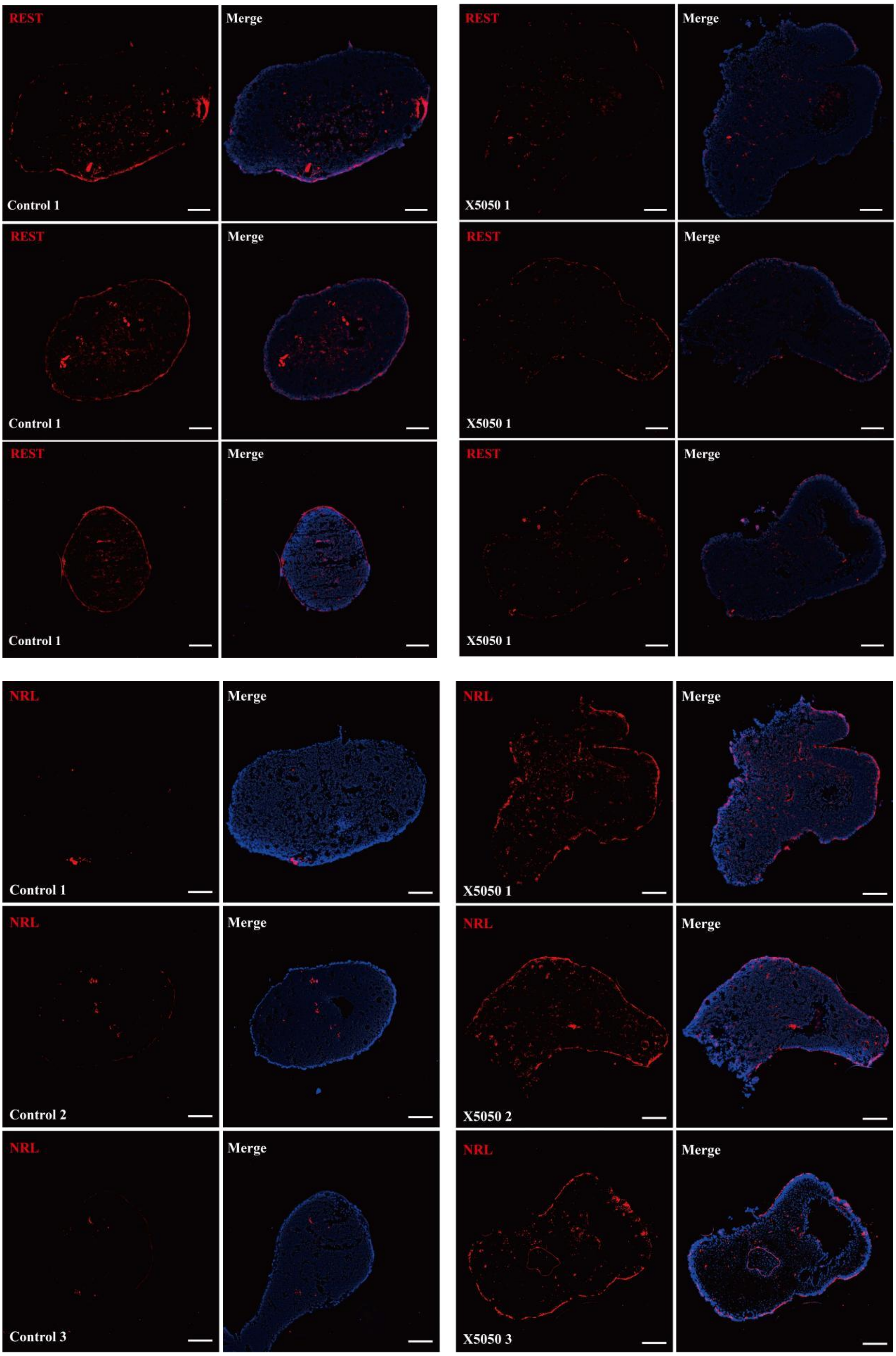
REST
Merge
Control 1
REST
Merge
X5050 1
REST
Merge
Control 1
REST
Merge
X5050 1
REST
Merge
Control 1
REST
Merge
X5050 1
NRL
Merge
Control 1
NRL
Merge
X5050 1
NRL
Merge
Control 2
NRL
Merge
X5050 2
NRL
Merge
Control 3
NRL
Merge
X5050 3

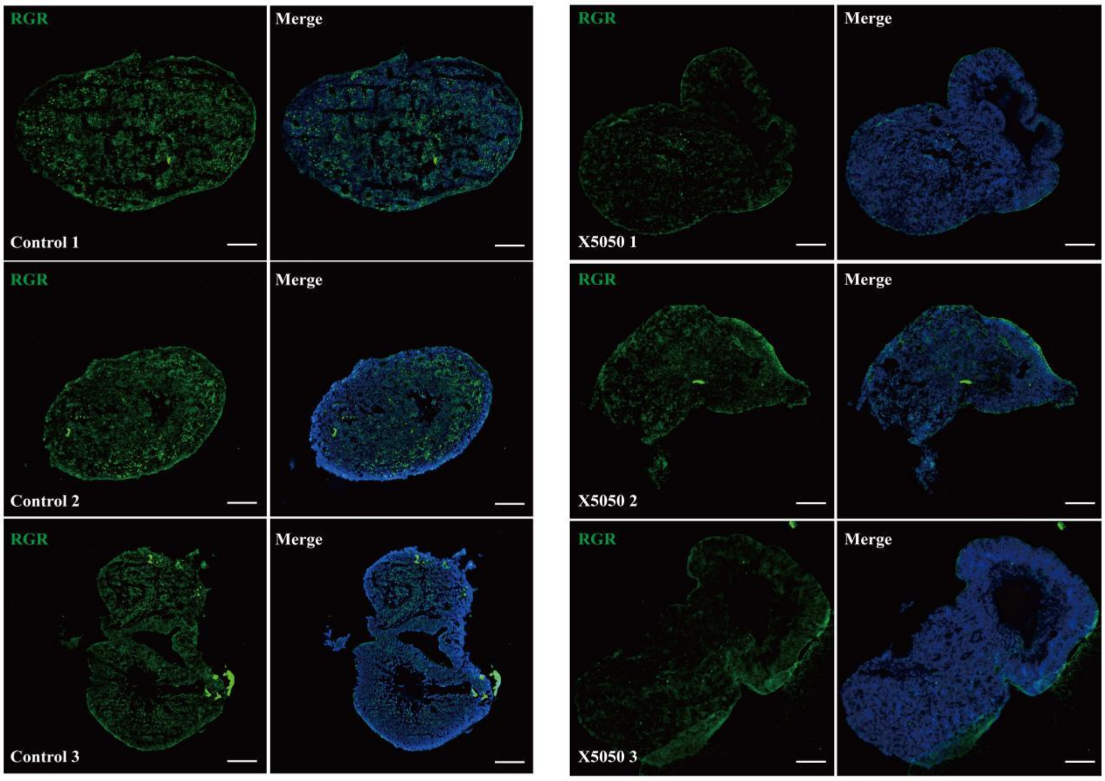

Figure S10. Immunofluorescence staining of REST, NRL and RGR in control and X5050-treated fetal retinospheres after 48 h. Scale bar, 100 µm.

Supplementary Tables

Table S1. Detailed information of samples in scRNA-seq.

Details / PCW	Estimated number of cells	Mean reads per cell	Median genes per cell	Sample Type	Sample origin
**PCW9**	15,831	25,368	2,018	Whole eyeball	China
**PCW10**	9,024	77,612	3,235	Whole eyeball	China
**PCW11**	6,535	43,909	2,560	Whole eyeball	China
**PCW14**	9,913	66,003	2,510	Whole eyeball	China
**PCW15**	9,532	30,383	1,664	Whole eyeball	China
**PCW18**	10,061	55,903	2,153	Whole eyeball	China
**PCW20**	11,910	37,180	2,086	Whole eyeball	China
**PCW23**	6,305	56,070	1,717	Whole eyeball	China
**PCW26-Neuroretina**	7,224	48,622	2,019	Whole retina	China
**PCW26-Others**	7,192	44,029	2,136	Others	China

Table S2. Marker genes for annotation.

Cell type	Marker genes
Retinal progenitor cells	*CCND1, HES1, SOX2*
Neurogenic retinal progenitor cells	*ATOH7, HES6, DLL3*
Retinal ganglion cells	*GAP43, SNCG, NEFM, NEFL, POU4F2*
Horizontal cells	*ONECUT2, ONECUT1*
Amacrine cells	*GAD1, GAD2, TFAP2A, TFAP2B*
Cone photoreceptor cells	*ARR3, PDE6H, GNAT2, PRDM1, THRB*
Rod photoreceptor cells	*NRL, RHO, RCVRN, NR2E3*
Bipolar cells	*PCP2, VSX1, TMEM215*
Müller glial cells	*RLBP1, RGR, GPX3*
Endothelium cells	*PECAM1, CLDN5, ENG*
Fibroblasts	*DCN, FBLN1, MGP*
Melanocytes	*MLANA, PMEL, TYRP1, DCT*
Corneal epithelium cells	*AQP3, AQP5, KRT14, KRT5, KRT7*
Macrophages	*C1QA, C1QB, C1QC, MRC1, MARCO*
Microglia	*P2RY12, P2RY13, CX3CR1, TMEM119*
Retinal pigment epithelium	*RPE65, BEST1, TTR, TYR, MITF*
Lens cells	*CRYGC, BFSP1, BFSP2, LIM2*

Table S3. Number of all cell types in scRNA-seq.

PCW / Clusters	PCW9	PCW10	PCW11	PCW14	PCW15	PCW18	PCW20	PCW23	PCW26-Neuroretina	PCW26-Others	Total
**RPCs**	4235	2417	1510	2445	3136	1675	3091	1133	214	161	20017
**NPCs**	853	519	411	557	700	559	763	367	262	253	5244
**MGCs**	0	0	0	1	2	22	6	144	147	45	367
**Rods**	291	152	152	420	1030	1404	2241	2192	1760	1542	11184
**HCs**	330	448	443	958	142	526	394	35	256	130	3662
**BCs**	223	134	64	175	322	476	363	668	1395	721	4541
**ACs**	382	288	424	636	434	746	539	124	214	165	3952
**RGCs**	1950	1958	1327	618	395	448	702	170	114	147	7829
**Fibroblasts**	1478	438	771	825	550	769	436	66	72	1921	7326
**Melanocytes**	2084	451	124	165	84	119	76	43	6	232	3384
**RPE**	194	52	20	42	22	11	7	1	5	65	419
**Cones**	135	145	153	327	580	470	245	117	158	80	2410
**Lens**	308	144	50	146	198	123	179	11	12	16	1187
**Macrophages**	54	14	25	52	22	105	83	13	5	229	602
**Microglia**	13	6	11	15	11	17	17	9	17	17	133
**Corneal epithelium**	7	0	5	0	2	0	1	1	0	0	16
**Endothelium**	32	12	18	61	4	24	11	10	9	72	253

Table S4. Number of all cell types in scATAC-seq.

PCW / Clusters	PCW9	PCW10	PCW11	PCW11-Rep	PCW14	PCW15	PCW18	PCW19	PCW20	PCW23	PCW26-Neuroretina	PCW26-Others	Total
**HCs**	204	384	636	1461	552	136	778	97	113	39	62	82	4544
**RGCs**	474	1721	1942	2927	690	306	285	115	177	12	40	103	8792
**ACs**	62	138	233	479	296	350	497	151	751	81	27	48	3113
**NPCs**	370	357	507	1028	356	1709	626	258	362	158	10	25	5766
**RPCs**	596	970	1420	2878	1179	3597	2263	926	908	660	38	61	15496
**Cones**	13	58	209	964	284	1446	494	219	120	44	78	40	3969
**Rods**	7	3	10	131	97	1519	2159	260	2754	1757	443	381	9521
**BCs**	22	100	130	479	625	1093	1670	433	1513	996	260	229	7550
**MGCs**	0	0	1	1	0	1	14	3	3	14	1	0	38
**Endothelium**	5	6	10	37	4	5	38	2	1	3	0	69	180
**Fibroblasts**	470	326	725	1778	192	716	902	228	36	55	10	3090	8528
**Melanocytes**	1114	711	322	1356	150	315	291	179	82	69	65	529	5183
**Macrophages**	28	12	40	287	38	38	86	17	25	14	2	402	989
**Microglia**	0	0	0	8	1	0	0	0	0	0	1	1	11
**RPE**	14	30	13	582	14	9	6	8	2	2	1	17	698
**Lens cells**	18	10	40	98	27	86	22	27	415	8	38	30	819

Table S5. Total number of cells in RPC subpopulations in scRNA-seq.

PCW / Clusters	PCW9	PCW10	PCW11	PCW14	PCW15	PCW18	PCW20	PCW23	PCW26-Neuroretina	PCW26-Others	Total
**RPC1**	2565	1678	100	32	18	12	5	6	1	11	4428
**RPC2**	0	0	12	25	1	1183	2532	19	187	84	4043
**RPC3**	0	5	1044	1752	0	47	4	0	0	0	2852
**RPC4**	701	420	249	296	85	247	416	64	6	7	2491
**RPC5**	0	1	0	3	2347	27	9	3	1	2	2393
**RPC6**	969	311	101	320	142	93	104	25	15	26	2106
**RPC7**	0	0	0	0	0	0	0	1010	0	0	1010
**RPC8**	0	2	4	17	543	65	21	6	4	5	667
**RPC9**	0	0	0	0	0	1	0	0	0	26	27

Table S6 Total number of cells in NPC subclusters in scRNA-seq.

PCW / Subclusters	PCW9	PCW10	PCW11	PCW14	PCW15	PCW18	PCW20	PCW23	PCW26-Neuroretina	PCW26-Others	Total
**NPC1**	1	36	68	188	2	380	583	20	28	43	1349
**NPC2**	54	103	154	246	96	107	92	17	5	14	888
**NPC3**	302	171	116	104	23	57	72	1	4	0	850
**NPC4**	496	209	72	11	0	1	0	0	0	0	789
**NPC5**	0	0	1	6	577	6	8	0	0	0	598
**NPC6**	0	0	0	2	0	6	8	0	225	196	437
**NPC7**	0	0	0	0	2	2	0	329	0	0	333
